# Chromatograms and Mass Spectra of High-Mannose and Paucimannose *N*-Glycans for Rapid Isomeric Identifications

**DOI:** 10.1021/acs.jproteome.3c00640

**Published:** 2024-02-16

**Authors:** Chia Yen Liew, Jien-Lian Chen, Yen-Ting Lin, Hong-Sheng Luo, An-Ti Hung, Bryan John Abel Magoling, Hock-Seng Nguan, Charles Pin-Kuang Lai, Chi-Kung Ni

**Affiliations:** † Institute of Atomic and Molecular Sciences, Academia Sinica, Taipei 106216, Taiwan; ‡ International Graduate Program of Molecular Science and Technology, National Taiwan University, Taipei 106216, Taiwan; § Molecular Science and Technology, Taiwan International Graduate Program, Academia Sinica, Taipei 106216, Taiwan; ∥ Department of Chemistry, National Taiwan Normal University, Taipei 116059, Taiwan; ⊥ Department of Chemistry, National Tsing Hua University, Hsinchu 300044, Taiwan; # Institute of Biochemical Sciences, College of Life Science, National Taiwan University, Taipei 106216, Taiwan; ∇ Chemical Biology and Molecular Biophysics Program, Taiwan International Graduate Program, Academia Sinica, Taipei 115201, Taiwan; ○ Genome and Systems Biology Degree Program, National Taiwan University and Academia Sinica, Taipei 106216, Taiwan

**Keywords:** high-mannose *N*-glycans, structure, logically derived sequence tandem mass spectrometry, LODES/MS*^n^*, CID spectra, chromatogram, bovine whey protein, soybean protein, human mammary epithelial cell, human breast carcinoma cell

## Abstract

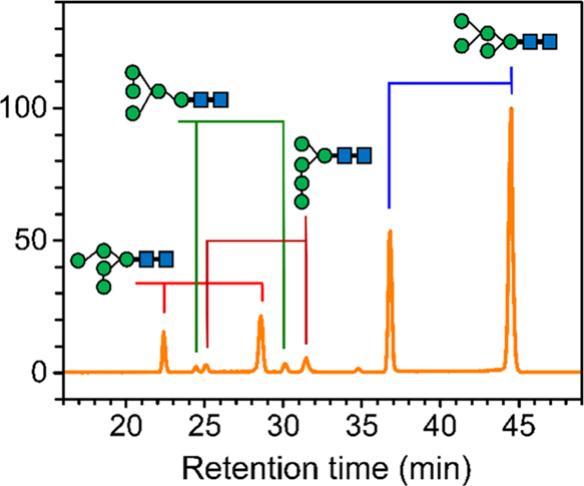

*N*-Linked glycosylation is one of the most essential post-translational modifications of proteins. However, *N*-glycan structural determination remains challenging because of the small differences in structures between isomers. In this study, we constructed a database containing collision-induced dissociation MS*^n^* mass spectra and chromatograms of high-performance liquid chromatography for the rapid identification of high-mannose and paucimannose *N*-glycan isomers. These *N*-glycans include isomers by breaking of arbitrary numbers of glycosidic bonds at arbitrary positions of canonical Man_9_GlcNAc_2_*N*-glycans. In addition, some GlcMan_n_GlcNAc_2_*N*-glycan isomers were included in the database. This database is particularly useful for the identification of the *N*-glycans not in conventional *N*-glycan standards. This study demonstrated the application of the database to structural assignment for high-mannose *N*-glycans extracted from bovine whey proteins, soybean proteins, human mammary epithelial cells, and human breast carcinoma cells. We found many *N*-glycans that are not expected to be generated by conventional biosynthetic pathways of multicellular eukaryotes.

## Introduction

One of the most important post-translational modifications of proteins is *N*-linked glycosylation. *N*-Linked glycans are involved in the stabilization of protein structures and the regulation of protein function.^1,2^ To fully understand protein stabilization and regulation, the structures of *N*-glycans must be characterized. For *N*-glycan structural determination, mass spectrometry has higher sensitivity compared with other methods such as nuclear magnetic resonance spectroscopy and enzyme digestion.^3,4^ Mass spectrometry is suitable for a small amount of samples, for example, the *N*-glycans extracted from cancer cells. Various mass spectrometry methods have been developed for *N*-glycan structural determination.^5−17^

A new MS method, namely, logically derived sequence (LODES) tandem mass spectrometry (MS*^n^*), for oligosaccharide structural determination has been developed recently.^18−24^ In LODES/MS*^n^*, oligosaccharide structures are determined using collision-induced dissociation (CID) in multistage tandem mass spectrometry.^25−29^ Unlike conventional mass spectrometry approaches, LODES/MS*^n^* does not require a mass spectrum library of oligosaccharides or *N*-glycan standards. Thus, this new method is particularly useful for the structural determination of *N*-glycans without standards. In this study, we used LODES/MS*^n^* to determine *N*-glycan structures and constructed a database of high-mannose and paucimannose *N*-glycans for rapid *N*-glycan isomer structural determination. The database included the chromatograms of high-performance liquid chromatography (HPLC) and multistage CID mass spectra. Databases of *N*-glycans have been constructed and reported by many research groups.^30−34^ The differences between our database and the databases reported in the literature are as follows: (1) our database consists of all possible high-mannose and paucimannose *N*-glycan isomers generated by breaking of arbitrary numbers glycosidic bonds at arbitrary positions of the canonical Man_9_GlcNAc_2_*N*-glycan. These *N*-glycans are not limited to the available *N*-glycan standards or the isomers according to the conventional biosynthetic pathways of multicellular eukaryotes. In addition, some GlcMan*_n_*GlcNAc_2_*N*-glycan isomers were included in the database. (2) We used intact *N*-glycans in our database. Permethylation, reduction, or labeling at the reducing end of *N*-glycans is not required. Therefore, the potential interference from the products generated by side reactions during permethylation, reduction, or labeling is completely eliminated. Because both α- and β-anomeric configurations of GlcNAc at the reducing end of intact *N*-glycans coexist in solution, the chromatogram exhibits two peaks for each intact *N*-glycan isomer when α- and β-anomers are separated by high-performance liquid chromatography (HPLC). Although the chromatogram may be complex when two peaks appear for one isomer, two peaks of each isomer provide two opportunities to identify the structure, greatly reducing any potential errors in structural assignment.

## Experimental Methods

### Sources of Materials

The sources of materials are listed in Section(A) in the Supporting Information. Extraction of IgY from hen egg yolk and membrane proteins from human cell lines has been described in previous studies.^24,35,36^

### *N*-Glycans Released through Ammonia-Catalyzed Reaction

*N*-Glycans were released from proteins through ammonia-catalyzed reactions, as described in a previous study.^37^ In brief, protein powder was dissolved in 25% ammonia aqueous solution for a 16 h reaction at 60 °C. After the reaction, the ammonia in the solution was removed using a rotary evaporator, and proteins were removed through ethanol precipitation. The released *N*-glycans were purified by the removal of residual proteins through a C18 cartridge, followed by the removal of potential contaminants and salt through a nonporous graphitized carbon (NPGC) cartridge and size exclusion chromatography.

### *N*-Glycans Released from Soybean Proteins by Using PNGase F

*N*-Linked glycans were released from soybean proteins through reactions with PNGase F, which were conducted in a solution consisting of 50 mM sodium phosphate (pH 7.5), with 24 h incubation at 37 °C. The released glycans were purified through ethanol precipitation to remove proteins, followed by solid-phase extraction by using a C18 cartridge to further remove residual proteins and an NPGC cartridge to remove salts.

### *N*-Glycans Released from Human Cell Lines

Cell membrane proteins were extracted using the differential centrifugation protocol adopted from Li et al.^35^ Denaturing buffer (500 μL) containing 5% SDS was used to suspend the membrane protein pellet, and the solution was heated at 100 °C for 10 min, followed by cooling at 0 °C for 10 min. To release *N*-glycans from the proteins, 50 μL of PNGase F solution (250,000 units), 100 μL of 10× GlycoBuffer, 100 μL of 10% NP-40, and 300 μL of deionized water were added and incubated at 37 °C overnight. The released *N*-glycans were purified through ethanol precipitation. After centrifugation, the supernatant was completely dried down in a centrifugal concentrator to remove the ethanol prior to solid-phase extraction by using C18 and NPGC cartridges.

### Enzymatic Degradation of Large *N*-Glycans

Some *N*-glycans were generated by the degradation of large *N*-glycan standards purchased from Omicron Biochemicals, Inc., by using the enzyme α-mannosidase from *Canavalia ensiformis* and α-1–6 mannosidase. The degradation reaction conditions were maintained according to the manufacturer’s protocols. In brief, 1 μL of high-mannose *N*-glycan standard (1 mM) was added to the reaction buffer. The reaction mixture for α-mannosidase from *C. ensiformis* contained 15 μL of DI water, 2 μL of GlycoBuffer 4 (10×), and 2 μL of zinc (10×), whereas the reaction mixture for α-1–6 mannosidase contained 15 μL of DI water, 2 μL of GlycoBuffer 1 (10×), and 2 μL of BSA (100 μg/mL). The reaction mixture was incubated at 37 °C with shaking at 800 rpm for 5 min before 0.1 μL of α-mannosidase from *C. ensiformis* or α1–6 mannosidase was added. To obtain the desired high-mannose *N*-glycans, mannosidase was removed from the reaction mixture using 0.6 μL of ZipTip C4 (Merck, Ltd., Taipei, Taiwan) at different time points.

### Two-Dimensional HPLC Separation

Two-dimensional HPLC was used to separate *N*-glycans. A CM 5000 series HPLC (Chromaster, Hitachi, Chiyoda-ku, Tokyo, Japan) with a TSKgel amide-80 column (150 mm × 2.0 mm, particle size of 5 μm, Tosoh Bioscience GmbH, Griesheim, Germany) was used for the first-dimension separation, and a fraction collector (FC204, Gilson, Middleton, WI) was used for fraction collection. The mobile phases used in HPLC were deionized water (solution A) and HPLC-grade acetonitrile (solution B), and the conditions for the TSKgel amide-80 column were as follows: the flow rate was 0.2 mL/min; the gradient was changed linearly from *A* = 35% and *B* = 65% at *t* = 0 to *A* = 45% and *B* = 55% at *t* = 50 min.

The fractions collected from the first HPLC eluents were injected into the second HPLC instrument (Chromaster, Hitachi, Chiyoda-ku, Tokyo, Japan) with a Hypercarb column (2.1 mm × 150 mm or 2.1 mm × 100 mm, particle size of 3 μm, Thermo Fisher Scientific, Waltham, MA) for the second-dimension separation. The HPLC conditions for the Hypercarb column were as follows: the flow rate was 0.2 mL/min; the gradient was changed linearly from *A* = 92% and *B* = 8% at *t* = 0 to *A* = 82% and *B* = 18% at *t* = 30 min. A fraction collector (FC204, Gilson, Middleton) was used for fraction collection.

### Mass Spectrometry

Nanospray Flex housing (Thermo Fisher Scientific) coupled to a linear ion trap mass spectrometer (LTQ XL, Thermo Fisher Scientific) was used for nanoelectrospray ionization (nano-ESI) mass spectrometry. Samples were prepared in a 50:50 (v/v) water/methanol mixture at a concentration of 5 × 10^–5^ M with NaCl (5 × 10^–5^ M), and 2 μL of each sample was loaded into a borosilicate glass nano-ESI emitter. The settings of the mass spectrometer were as follows: a nano-ESI source voltage of 1.5 kV, a capillary voltage of 130 V, a heated capillary temperature of 120 °C, a tube lens voltage of 230 V, an activation *Q* value of 0.25, an activation time of 30 ms, and normalized collision energy of 30–40%. The number of ions was regulated by the injection time (10–20 ms) or automatic gain control (1 × 10^5^ for full scan and 1 × 10^4^ for MS*^n^*). The precursor ion isolation width was set to 1u. Helium gas was used as a buffer gas for the ion trap as well as a collision gas in CID.

For HPLC-electrospray mass spectrometry, the chromatograms and MS*^n^* spectra were obtained using a heated electrospray ionization (HESI-II) probe with an Ion Max housing and a linear ion trap mass spectrometer (LTQ XL, Thermo Fisher Scientific) coupled to an HPLC system (Dionex Ultimate 3000, Thermo Fisher Scientific) with a Hypercarb column (2.1 mm × 150 mm, particle size of 3 μm, Thermo Fisher Scientific). The HPLC conditions for the Hypercarb column were as follows: the flow rate was 0.15 mL/min; the gradient was changed linearly from *A* = 100% and *B* = 0% at *t* = 0 to *A* = 93% and *B* = 7% at *t* = 5 min, and then to *A* = 80% and *B* = 20% at *t* = 60 min. The entire HPLC and mass spectrometer system was controlled using Dinoex Chromatography MS Link 2.14, Chromeleon Version 6.80 SR13, LTQ Tune Plus Version 2.7.0.1103 SP1, and Thermo Xcalibur 2.2 SP1.48 software from Thermo Fisher Scientific. The settings of the mass spectrometer were the same as those used in nanoelectrospray mass spectrometry, except that the ion spray voltage was 4.00 kV, the transfer capillary temperature was 280 °C, the capillary voltage was 80 V, and the tube lens voltage was 150 V. Dextran was used as reference in chromatogram.

## Results and Discussion

The sources of *N*-glycans for database construction included (1) commercial products, (2) *N*-glycans extracted and purified from biological samples, and (3) *N*-glycans enzymatically degraded from large *N*-glycans. The structures of commercially synthesized *N*-glycans have been identified by the manufacturer, and they were cross-checked using LODES/MS*^n^* in this study. *N*-Glycans extracted from biological samples were purified, followed by their structural determination using LODES/MS*^n^*; some of them were cross-checked using enzyme digestion.

These *N*-glycans were sent individually into a mass spectrometer coupled with an HPLC for recording the retention times and mass spectra. Meanwhile, dextran was used as a reference of retention time. The retention times of dextran were measured prior to (or right after) the measurement of each *N*-glycan, and the relative values of *N*-glycan retention times to dextran retention times, namely, dextran indexes, were calculated. Multiple measurements, which were carried out by three persons using three mass spectrometers in 2 years, were recorded to ensure reproducibility and to obtain the range of potential fluctuations. The database includes (1) chromatogram, (2) one MS^2^ spectrum, two MS^3^ spectra of each *N*-glycan, and MS^4^ spectra for some *N*-glycans, (3) dextran indexes of each *N*-glycan, and (4) diagnostic fragments for the isomers with close retention times. The chromatograms and mass spectra are in graphic format in the main text and are in numerical format deposited in MassIVE Repository. The diagnostic fragments are presented in numerical format in Section (D) in the Supporting Information.

### *N*-Glycan Structural Determination

**Figure 1:**
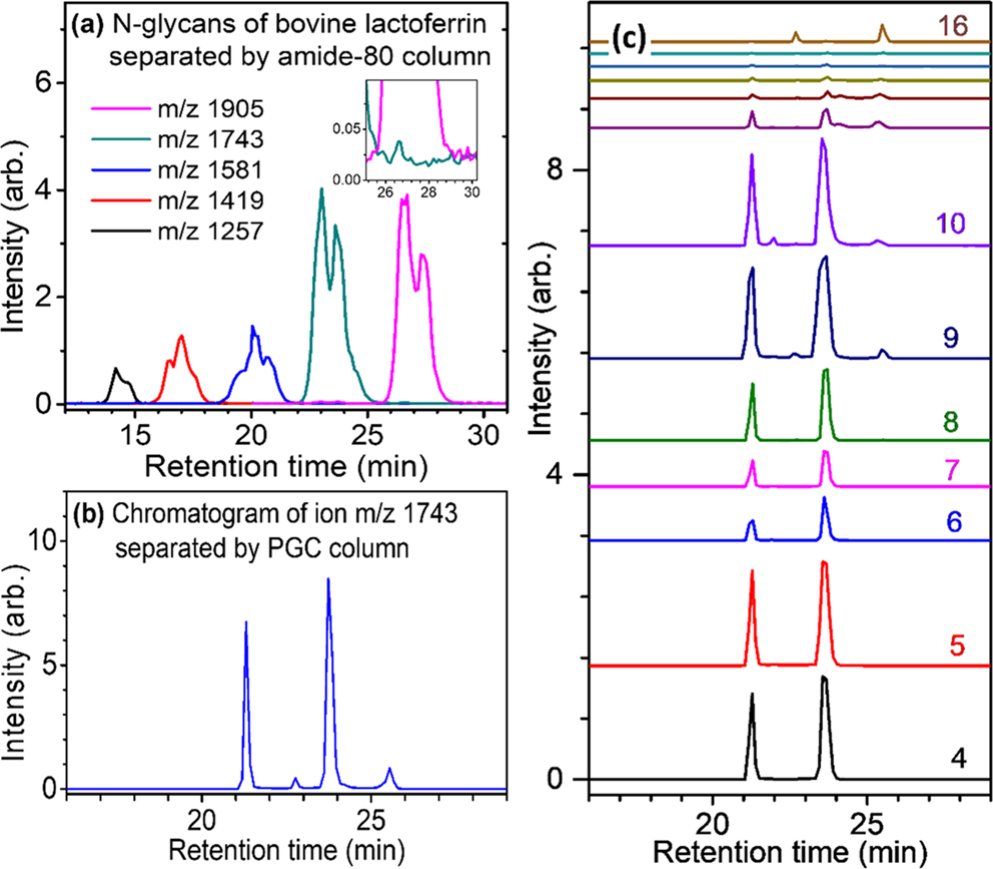
Chromatograms of *N*-glycans extracted from bovine lactoferrin. (a) Chromatogram of *N*-glycans separated using a TSKgel amide-80 column (150 mm × 2.0 mm, particle size of 5 μm). *m*/*z* values of 1905 (sodium ion adduct of Hex_9_GlcNAc_2_), 1743 (sodium ion adduct of Hex_8_GlcNAc_2_), 1581 (sodium ion adduct of Hex_7_GlcNAc_2_), 1419 (sodium ion adduct of Hex_6_GlcNAc_2_), and 1257 (sodium ion adduct of Hex_5_GlcNAc_2_). (b) Chromatogram (*m*/*z* 1743) of *N*-glycans using a Hypercarb PGC column (2.1 mm × 100 mm, particle size of 3 μm). These *N*-glycans were the eluents collected at retention time *t* = 22–25.5 min in the chromatogram (a). (c) Chromatograms (*m*/*z* 1743) of *N*-glycans using a Hypercarb PGC column (2.1 mm × 100 mm, particle size of 3 μm). These *N*-glycans were the eluents collected at retention time *t* = 20–26.5 min in chromatogram (b) for every 0.5 min. The numbers on the right-hand side represent the tube numbers of fraction collection.

In this study, we used bovine lactoferrin as an example to demonstrate how *N*-glycans were purified, and we used Hex_8_HexNAc_2_ isomers extracted from bovine lactoferrin as examples to demonstrate how *N*-glycan structures were determined using LODES/MS*^n^*. After *N*-glycans were released from lactoferrin through an ammonia-catalyzed reaction, *N*-glycans were purified by solid-phase extraction and size exclusion chromatography. These *N*-glycans were then sent into two-dimensional HPLC for further purification and isomer separation. A study demonstrated that approximately 1% of *N*-glycans undergo in-source decay and crack into small *N*-glycans during the ionization process of electrospray ionization.^38^ If a high concentration of large *N*-glycans is mixed with a low concentration of small *N*-glycans in the ESI solution, ESI in-source decay of large *N*-glycans may interfere with the structural identification of small *N*-glycans. To eliminate this potential interference, different sizes of *N*-glycans were separated through first-dimension HPLC by using a TSKgel amide-80 column. Figure 1a presents a chromatogram of various sizes of high-mannose *N*-glycans separated using the amide-80 column. The chromatogram exhibits small amounts of small *N*-glycans that overlap with large *N*-glycans in terms of the retention time, e.g., a small amount of Man_8_GlcNAc_2_ (*m*/*z* 1743) exists at a retention time of 26–27 min that Man_9_GlcNAc_2_ (*m*/*z* 1905) is the major component at the same retention time, as illustrated in the inset of Figure 1a. This Man_8_GlcNAc_2_ is likely produced by the ESI in-source decay of Man_9_GlcNAc_2_; thus, the Man_8_GlcNAc_2_ at the aforementioned retention time was not analyzed, and only the fraction of Man_8_GlcNAc_2_ collected at the retention time from 22 to 25.5 min was analyzed. Each fraction collected from the eluents of the TSKgel amide-80 column contained *N*-glycans of a given size but may also contain more than one isomer.

The samples obtained from the eluents of the TSKgel amide-80 column were concentrated and then subjected to isomer separation on the HPLC instrument with a PGC column. As an example, the chromatogram of the *N*-glycans collected at retention time *t* = 22–25.5 min from the TSKgel amide-80 column eluents is illustrated in Figure 1b. The chromatogram exhibits four peaks for the ion with an *m*/*z* value of 1743, representing the sodium adduct of Hex_8_HexNAc_2_ isomers separated using the PGC column. The PGC column is known for separating anomeric isomers; therefore, the four peaks in Figure 1b might be attributed to the α- and β-anomers of two isomers. To verify this hypothesis, the eluents from the PGC column were collected as fractions every 0.5 min so that the compound corresponding to each peak in Figure 1b was collected into different tubes. The same collection procedure was repeated several times such that the eluents with the same retention time were collected into the same tube. These eluents were stored at room temperature for 6 h before they were concentrated and reinjected into the same PGC column individually. If two peaks in Figure 1b are from the same isomer and they only differ by the anomericity at the reducing end, the reinjection of the eluents into the same PGC column will result in two peaks again in the chromatogram, and the relative intensities and retention times of these two peaks must remain the same as those in Figure 1b. This is because α- and β-anomers undergo mutarotation to transform into each other, and it takes 30 min to 2 h in solution at room temperature to reach equilibrium. Although each fraction contains only one anomer initially when the anomer is collected from the PGC eluents, the anomer undergoes mutarotation during the storage time. Consequently, two anomers are present in each collected fraction. The chromatograms of these eluents reinjected into the PGC column, illustrated in Figure 1c, exhibit peaks at retention times, *t* = 21.3 and 23.8 min, which can be attributed to one isomer, and peaks at retention times, *t* = 22.7 and 25.5 min, which can be attributed to the other isomer. The samples in tubes 5, 9, and 16 have high intensity because they correspond to the fractions collected at retention times, t = 21.3, 23.8, and 25.5 min, respectively [Figure 1b].

**Figure 2:**
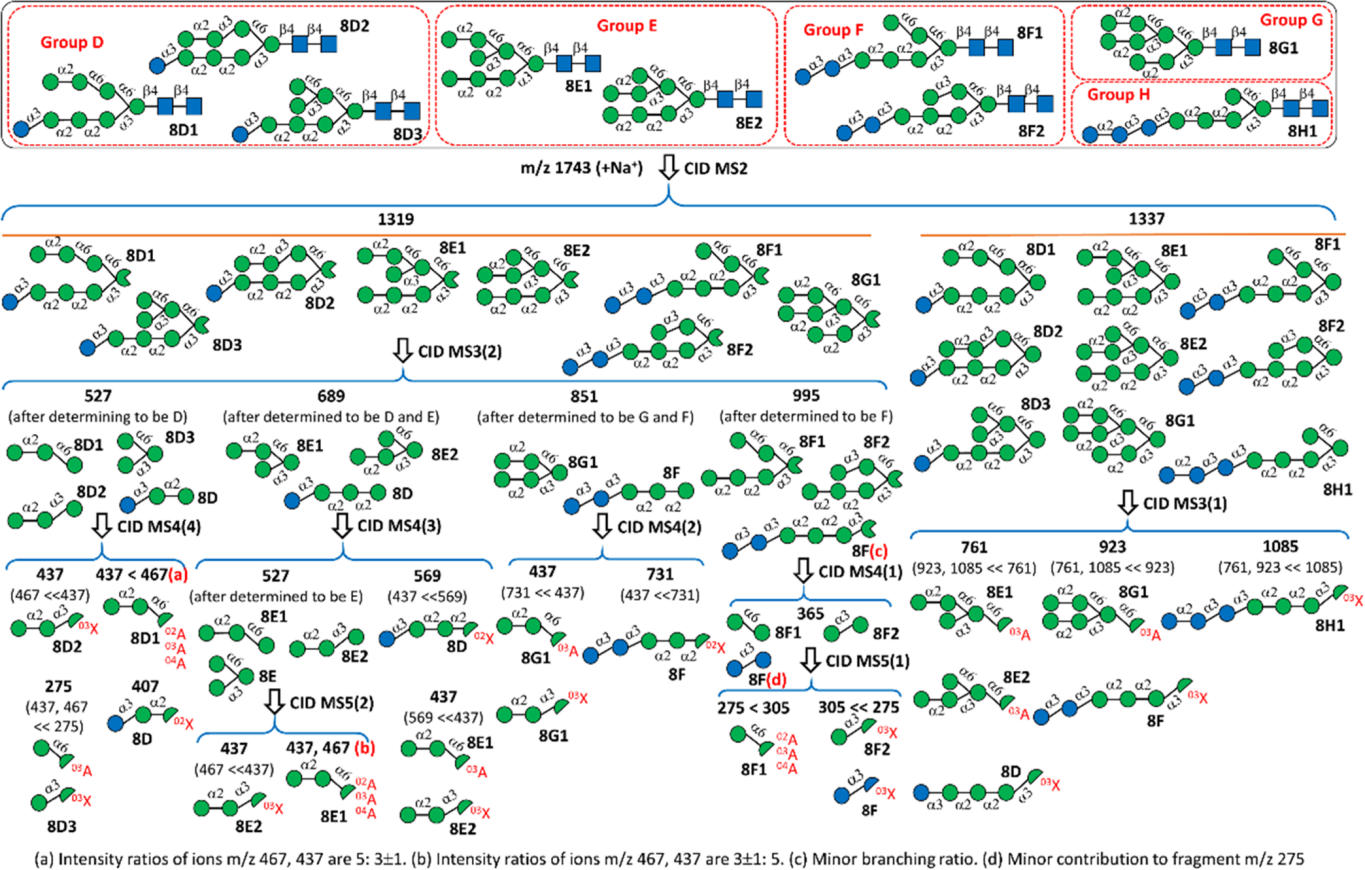
CID sequences of sodium ion adducts, derived from LODES, used to differentiate between Hex_8_GlcNAc_2_ isomers. Three-quarter circle and half-circle represent dehydration and cross-ring dissociation, respectively.

Tubes 4–8 contained only one isomer of Hex_8_HexNAc_2_, and tube 16 contained the other isomer of Hex_8_HexNAc_2_. The eluents in tubes 4–8 and tube 16 were subjected to mass spectrometry separately for structural determination. Figure 2 illustrates the CID sequences used to differentiate between Hex_8_GlcNAc_2_ isomers. These sequences were based on the collision-induced dissociation mechanism of carbohydrate sodium ion adducts.^26−29^ These mechanisms can be summarized as three propensities: (1) Dehydration mainly occurs at the reducing end. (2) Cross-ring dissociation mainly occurs at the reducing end and follows the rule of retro-aldol reaction. (3) Cleavage of glycosidic bonds occurs at any position. Details of the applications of LODES to determine the *N*-glycans have been reported in our previous report.^23^ In brief, Hex_8_GlcNAc_2_ isomers are classified into five groups, namely, D, E, F, G, and H, as illustrated in Figure 2. These isomers include all isomers produced by breaking of arbitrary numbers of glycosidic bonds at arbitrary positions of Glc_3_Man_9_GlcNAc_2_, an *N*-glycan that is transferred from dolichol-phosphate to proteins before removal of any Glc or Man by enzyme. The structural determination starts from the mass spectrum through the CID sequence 1743 → 1337 → fragments [MS^2^ → MS^3^(1) on the right-hand side of Figure 2], which classifies the isomers into three categories, (D and E), (F and G), and H, depending on the fragments *m*/*z* 761, 923, or 1085 found in the spectrum, respectively. The mass spectrum through the CID sequence 1743 → 1319 → 689 → fragments [MS^2^ → MS^3^(2) → MS^4^(3) in Figure 2(middle)] distinguishes the isomers between groups D and E: fragment *m*/*z* 569 or 437 found in this CID indicates isomers belonged to groups D or E, respectively. The identification of isomers in groups D and E is made through the CID sequences, 1743 → 1319 → 689 → 527 → fragments and 1743 → 1319 → 527 → fragments, respectively. The mass spectrum through the CID sequence 1743 → 1319 → 851 → fragments [MS^2^ → MS^3^(2) → MS^4^(2) in Figure 2(middle)] distinguishes the isomers between groups F and G: fragment *m*/*z* 437 or 731 found in this CID indicates isomers belonged to group F or G, respectively. The identification of isomers in group F is made through the CID sequence 1743 → 1319 → 995 → 365 → fragments.

**Figure 3:**
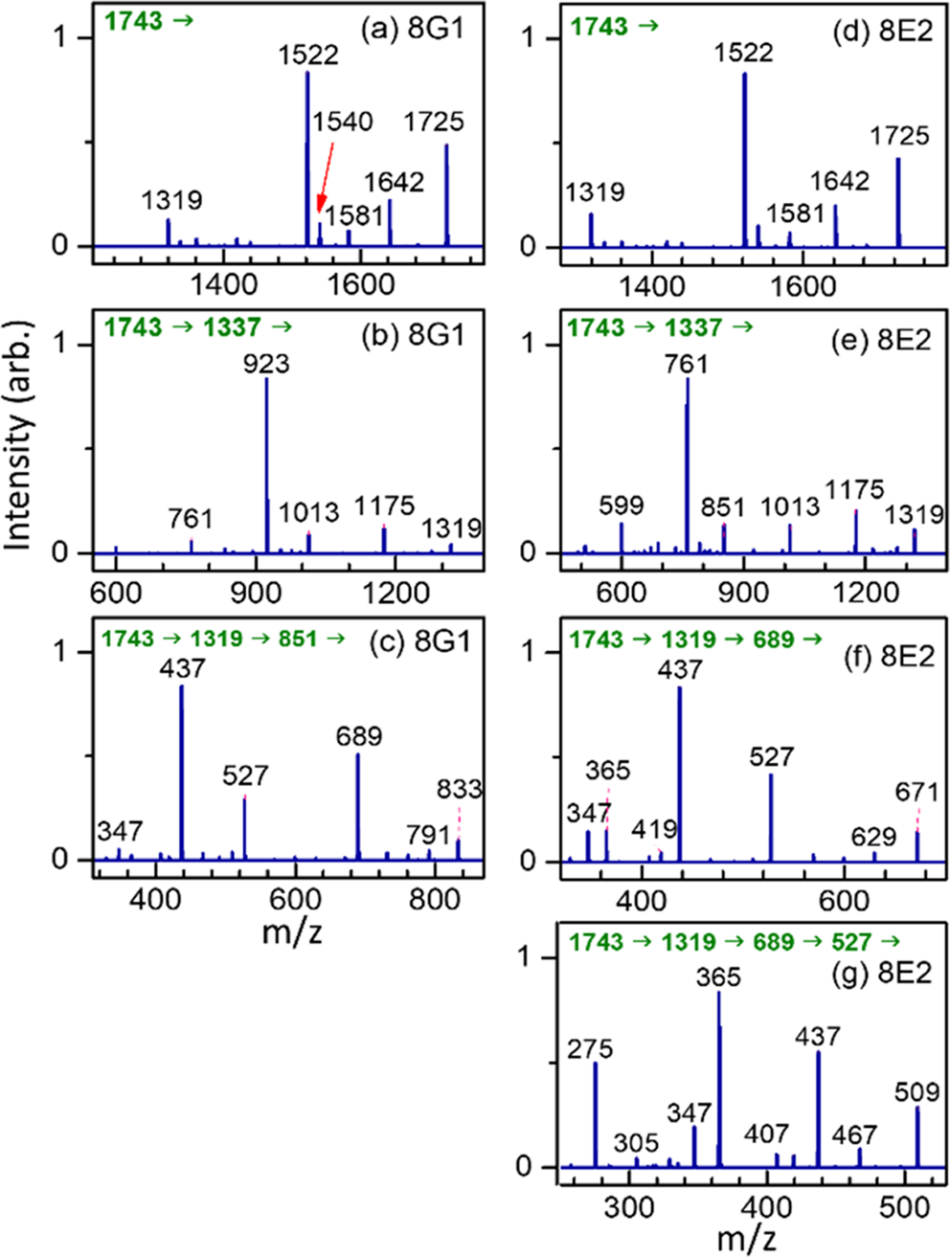
CID spectra of sodium ion adducts for the structural determination of two Hex_8_GlcNAc_2_ isomers extracted from bovine lactoferrin; (a–c): isomer 8G1, (d–g): isomer 8E2.

Figure 3a–c illustrates the CID spectra of the isomers in tubes 4–8. The precursor ion, sodium ion adduct of Hex_8_GlcNAc_2_, had an *m*/*z* value of 1743, and Figure 3a shows the MS^2^ spectrum based on the CID sequence, namely, 1743 → fragments. The main fragments were those resulting from dehydration at the reducing end (loss of *m* = 18 Da, resulting B ion *m*/*z* 1725), cross-ring dissociation at the reducing end (loss of *m* = 101 Da, resulting ^0,2^A ion *m*/*z* 1642), loss of one hexose through glycosidic bond cleavage (loss of *m* = 162 Da, resulting Y ion *m*/*z* 1581), loss of GlcNAc at the reducing end through glycosidic bond cleavage (loss of *m* = 203 Da, resulting C ion *m*/*z* 1540), and loss of GlcNAc at the reducing end through glycosidic bond cleavage (loss of *m* = 221 Da, resulting B ion *m*/*z* 1522), where the notations A, B, C, and Y are used according to the fragment nomenclature of Domon and Costello.^39^ We found that these dissociation channels produced the major fragment ions in the CID MS^2^ spectra of all high-mannose and paucimannose *N*-glycans. Although MS^2^ spectra have the highest signal-to-noise ratio among all MS*^n^* spectra, most major fragments produced from the *N*-glycan core structure are identical for most *N*-glycans. Thus, most *N*-glycan isomers have similar MS^2^ spectra, as demonstrated in Figure 3a,d, in the CID spectra of the database in the following section, and in Appendix Section. Some features of MS^2^ spectra are different between isomers, as discussed in the next section, but these features mainly result from minor dissociation channels.

The MS^3^ spectrum, Figure 3b, obtained based on the CID sequence of 1743 → 1337 → fragments, classified isomers into three groups, namely, (D and E), (F and G), and H, by comparing the intensities of fragment ions with *m*/*z* values of 761, 923, and 1085 according to LODES illustrated in Figure 2. In this CID sequence, ion *m*/*z* 1337 is the C ion (denoted as C_*n*–2_ ion) obtained by the cleavage of β-Man-(1 → 4)-GlcNAc linkage. Figure 3b shows that the intensity of ion *m*/*z* 923 is much higher than that of ions *m*/*z* 761 and 1085, indicating that this *N*-glycan belongs to the (F and G) group. To further distinguish between F and G groups, the MS^4^ spectrum based on the CID sequence of 1743 → 1319 → 851 → fragments was used [Figure 3c, according to the CID sequence of MS^2^ → MS^3^(2) → MS^4^(2) in Figure 2]. In this CID sequence, ion *m*/*z* 1319 is the B ion (denoted as B_*n*–2_ ion) obtained by breaking the β-Man-(1 → 4)-GlcNAc linkage. A comparison of fragment ions with *m*/*z* values 437 and 731 in the MS^4^ spectrum revealed that the intensity of the ion with an *m*/*z* value of 437 was much higher than that of the ion with an *m*/*z* value of 731, indicating that the *N*-glycan belonged to the G group. There is only one isomer in group G; therefore, we concluded that this *N*-glycan is 8G1.

The structure of the other isomer collected in tube 16 was similarly analyzed. The high intensity of ion *m*/*z* 761 in Figure 3e indicates that the isomer belonged to groups D or E according to the CID sequence of MS^2^ → MS^3^(1) in Figure 2. As presented in Figure 3f, the intensity of the ion with an *m*/*z* value of 437 was much higher than that of the ion with an *m*/*z* value of 569, indicating that the isomer belonged to group E according to the CID sequence of MS^2^ → MS^3^(2) → MS^4^(3) in Figure 2. Finally, the high intensity of the ion with an *m*/*z* value of 437 relative to that of the ion with an *m*/*z* value of 467 in Figure 3g suggests the isomer is 8E2 according to the CID sequence of MS^2^ → MS^3^(2) → MS^4^(3) → MS^5^(2) in Figure 2.

For paucimannose *N*-glycans, MS^3^ or MS^4^ CID spectra were used to determine the structures. These CID spectra can be obtained using HPLC/ESI/MS through real-time online measurement if the concentrations of *N*-glycans are not very low. In contrast, MS^4^ or MS^5^ CID spectra are required for the structural determination of high-mannose *N*-glycans. The measurement of MS^4^ and MS^5^ CID spectra with good signal-to-noise ratios takes typically 2–5 min, which is longer than the duration (approximately 20–30 s) of a peak appearing in a chromatogram. Most MS^4^ and MS^5^ CID spectra of high-mannose *N*-glycans cannot be obtained using HPLC/ESI/MS through real-time online measurement. In this study, we collected the fractions of the HPLC eluents, followed by structural analysis by using nano-ESI/MS for high-mannose *N*-glycans. Parts of the CID spectra have been reported in our previous study,^36^ and the rest of the CID spectra are illustrated in Section(C) in the Supporting Information.

### Construction of the Chromatogram and CID MS*^n^* Mass Spectrum Database

After we identified the structures of all *N*-glycans, we constructed a database for rapid *N*-glycan structural identification. The database only included HPLC chromatograms, one MS^2^ spectrum, and two MS^3^ CID spectra, which were obtained from HPLC/ESI/MS through real-time online measurement. The database is applicable for rapid *N*-glycan isomer structural identification. In few cases where the retention times of two isomers were very close and the MS^2^ and two MS^3^ CID spectra of these two isomers were similar, the MS^4^ CID spectra were included in the database.

#### ManGlcNAc_2_ and Man_2_GlcNAc_2_

**Figure 4:**
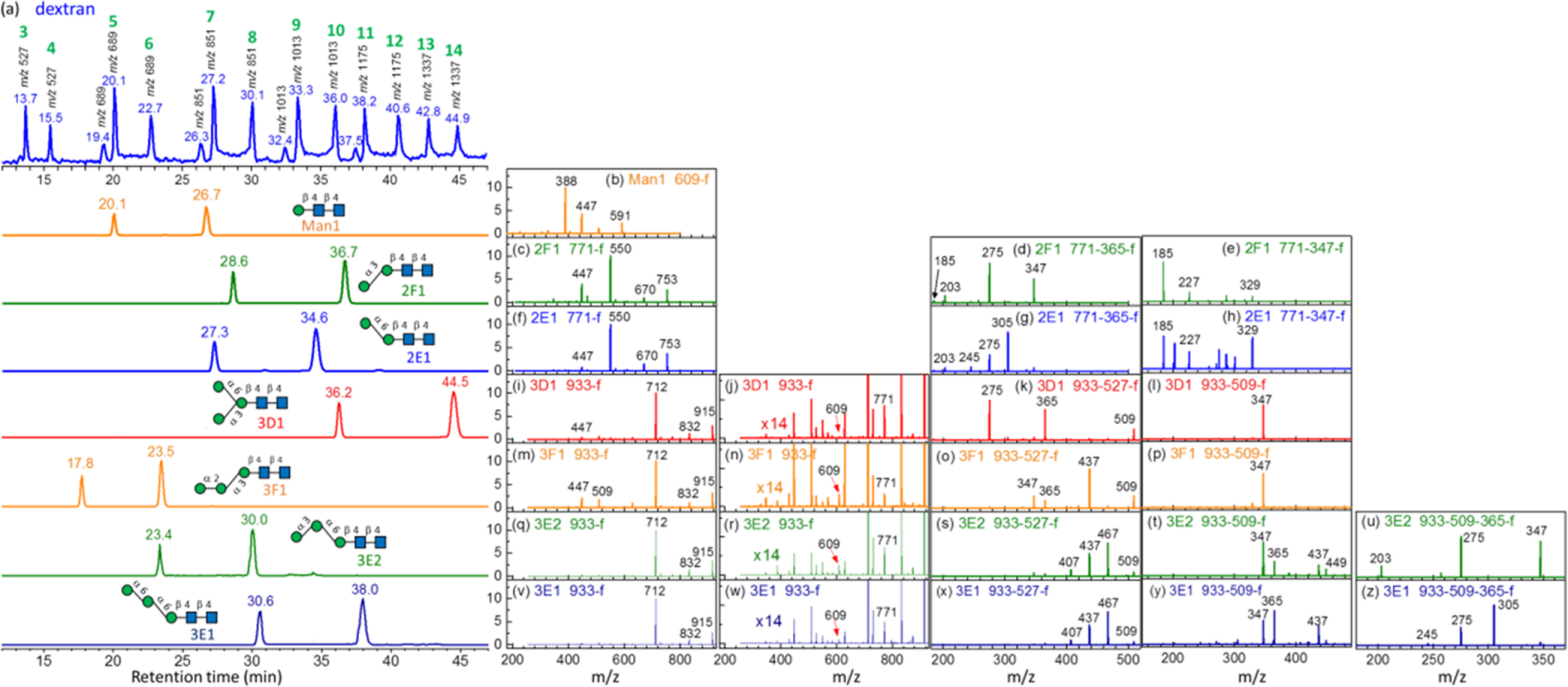
Database of Man_n_GlcNAc_2_, *n* = 1, 2, 3. (a) Chromatograms of dextran and paucimannose *N*-glycan Man*_n_*GlcNAc_2_, *n* = 1, 2, 3. The index numbers are represented in green at the top of each peak of dextran. (b–t) CID spectra of Man*_n_*GlcNAc_2_ sodium ion adducts. The CID sequences are listed at the top of each spectrum. All *y*-axes represent intensity in arbitrary units. Man1, 2F1, 2E1, 3D1, and 3F1 were commercial products; 3E1 and 3E2 were generated by degradation of 4E2 and 4E3 by enzymes.

There is only one isomer of ManGlcNAc_2_, and two isomers of Man_2_GlcNAc_2_. All of them were purchased from Omicron Biochemicals, Inc. The chromatograms and CID spectra are provided in Figure 4a,b–h, respectively. The chromatogram of the dextran analytical standard [derived from *Leuconostoc mesenteroides* and with an average molecular weight of 1000] was used as a reference. Notably, this dextran was not reduced at the reducing end, and two major peaks were found for each molecular size of dextran. Isomers of Man_2_GlcNAc_2_ could be easily distinguished based on the retention time in chromatograms. They could also be identified based on the CID spectra, MS^2^ spectra [Figure 4c,f], or MS^3^ spectra [Figure 4d,g,e,h]. In the MS^3^ spectrum based on the CID sequence of the C_n–2_ ion (*m*/*z* 365) (771 → 365 → fragments), LODES predicted that the cross-ring dissociation of the C_*n*–2_ ion followed the retro-aldol reaction; isomer 2F1, in which C_*n*–2_ ion had a 1 → 3 linkage at the reducing end had a higher intensity of fragment *m*/*z* 275 compared with that of fragments *m*/*z* 245 and 305 [Figure 4d]. By contrast, the C_*n*–2_ ion of isomer 2E1 had a 1 → 6 linkage at the reducing end, and the intensity ratio of fragments *m*/*z* 305, 275, and 245 predicted by LODES was 5:3 ± 1:1 ± 0.5 [Figure 4g].

#### Man_3_GlcNAc_2_

There are four isomers of Man_3_GlcNAc_2_. Isomers 3D1 and 3F1 were purchased from Omicron Biochemicals, Inc. Isomers 3E1 and 3E2 were generated by the degradation of the Man_4_GlcNAc_2_ isomers 4E2 and 4E3 by α-mannosidase from *C. ensiformis* (Jack bean), respectively. The chromatograms and CID spectra are illustrated in Figure 4a,i–z, respectively. The LODES for structural determination of Man_3_GlcNAc_2_ isomers is illustrated in Section(B) in the Supporting Information. The retention time of these Man_3_GlcNAc_2_ isomers is very different and can be used solely to distinguish these isomers.

The major fragments in the MS^2^ spectra of these four isomers were similar, except for that of isomer 3F1; the intensities of the fragments *m*/*z* 447 and 509 were high. The fragments of low intensities in MS^2^, illustrated in Figure 4j,n,r,w, show that the intensity of fragment *m*/*z* 609 from isomer 3D1 is much smaller than the others. Fragment *m*/*z* 609 results from the loss of two mannoses which can be produced from the other isomers by breaking one glycosidic bond. However, it requires cleavage of two glycosidic bonds from isomer 3D1. Thus, the relative intensities of fragments *m*/*z* 609 and 771 can be used to differentiate 3D1 from the other isomers.

The structures of isomers 3D1 and 3F1 could be identified using a CID spectrum based on the CID sequence through the C_*n*–2_ ion (*m*/*z* 527; 933 → 527 → fragments) [Figure 4k,o]. Two CID spectra were required to determine the structures of isomers 3E1 and 3E2. One was the CID spectrum based on the CID sequence of 933 → 527 → fragments [Figure 4s,x] that identified the *N*-glycans belonging to group E, and the other was the CID spectrum based on the CID sequence of 933 → 509 → 365 → fragments [Figure 4u,z] that distinguished between isomers 3E1 and 3E2. All of the structural determination of these Man_3_GlcNAc_2_ isomers by the aforementioned CID spectra is based on the retro-aldol reaction. Notably, the intensities of the fragment ions produced from C_*n*–2_ ion (*m*/*z* 527) cross-ring dissociation according to the retro-aldol reaction were at least 20 times higher than those of fragment ions that did not follow the retro-aldol reaction. For example, for isomer 3D1, the intensity of the ion with an *m*/*z* value of 275 produced from the C_*n*–2_ ion was >20 times higher than that of ions with *m*/*z* values of 407, 437, and 467; for isomer 3F1, the intensity of the ion with an *m*/*z* value of 437 produced from the C_*n*–2_ ion was >20 times higher than that of ions with *m*/*z* values of 275, 407, and 467; and for isomers 3E1 and 3E2, intensities of ions with *m*/*z* values of 467, 437, and 407 produced from C_*n*–2_ ions were >20 times higher than that of the ion with an *m*/*z* value of 275.

The CID spectra through B_*n*–2_ ion (*m*/*z* 509), 933 → 509 → 365 → fragments, provided an additional method for distinguishing isomers 3E1 and 3E2 from isomers 3D1 and 3F1. All of the aforementioned CID spectra featured a high signal-to-noise ratio and were obtained through HPLC/ESI/MS real-time online measurement, and they were included in the database.

The rest of database, Hex*_n_*GlcNAc_2_ (*n* = 4–10), are illustrated in Appendix Section. The diagnostic fragments in the CID spectra that were used to differentiate isomers are listed in Table S1 of Section(D) in the Supporting Information.

In the HPLC chromatogram, we used the retention time of dextran as a reference. Because the reducing end of dextran is not reduced, there are two major peaks for a given molecular weight, i.e., two anomers of a give oligosaccharide [Glc-α-(1 → 6)]*_n_*-Glc-α and [Glc-α-(1 → 6)]*_n_*-Glc-β. Thus, the conventional glucose unit is not suitable here. We used sequential numbers (denoted as dextran index) to label the major peaks of dextran, as illustrated in Figure 4a. Calculations from retention time to dextran index are described in Section(E) in the Supporting Information. Table 1 lists the dextran indexes of all paucimannose and high-mannose *N*-glycans. The repeating measurements showed that the error bar of index is ±2% for small index (<7) and up to ±5% for large index (>11).

**Table 1:** Dextran Index of Paucimannose and High-Mannose *N*-Glycans

glycan	index	glycan	index	glycan	index	glycan	index	glycan	index	glycan	index
Man1	5.06	4D2	8.46	5F2	8.61	6H1	7.67	7F1	7.13	8D3	9.01
Man1	7.01	4D2	11.22	5F2	11.22	6H1	9.85	7F1	9.17	8D3	11.47
		4D1	11.33	5D2	5.76	6F1	7.88	7E1	6.90	8E2	7.45
2F1	7.60	4D1	15.14	5D2	7.40	6F1	9.91	7E1	8.32	8E2	9.66
2F1	10.31	4D3	6.45	5D1	7.13	6F2	11.11	7D3	7. 29	8G1	6.59
2E1	7.28	4D3	8.27	5D1	9.26	6F2	14.71	7D3	9.23	8G1	8.24
2E1	9.69	4E1	7.97	5E4	6.67	6D3	7.37	7G1	8.16	8E1	6.72
		4E1	10.22	5E4	8.55	6D3	9.31	7G1	10.55	8E1	8.32
3D1	10.17	4E2	7.94	5E2	9.84	6D1	7.50	7E2	7.61		
3D1	13.90	4E2	10.31	5E2	13.34	6D1	9.22	7E2	9.82	9E1	6.58
3F1	4.54	4E3	6.83	5E3	9.14	6D2	6.34	7D2	6.35	9E1	8.34
3F1	6.25	4E3	9.05	5E3	12.13	6D2	8.22	7D2	8.23	9D1	7.60
3E2	6.25	4F1	4.80	5E1	9.99	6G1	6.70	7D1	7.67	9D1	9.20
3E2	8.17	4F1	6.51	5E1	13.28	6G1	8.55	7D1	9.50	9D2	9.70
3E1	8.33			5F1	6.35	6E2	7.87			9D2	12.47
3E1	11.04			5F1	7.90	6E2	9.82				
						6E1	6.16			10D1	7.55
						6E1	7.79			10D1	9.52

### Applications to Determining High-Mannose *N*-Glycans Extracted from Biological Samples

The database can be applied to determine the structures of *N*-glycans extracted from biological samples. The extracted *N*-glycans were first separated through HPLC by using the amide-80 column according to their sizes to avoid interference from ESI in-source decay.^38^ The fractions collected from the eluents of the amide-80 column were injected individually into the PGC column in an HPLC coupled to a mass spectrometer for isomer separation and structural assignment. Structures of these *N*-glycans were determined using the following criteria: (1) Comparison of both α- and β-anomer dextran indices (retention times) of the ions with the indices of selected *m*/*z* values in the database. (2) Comparison of the relative intensities of α- and β-anomers of the ions with that of the selected *m*/*z* values in the database. Although the relative abundance of isomers varies in different biological samples, the relative ion intensities of two anomers of the same isomer must be the same as those in the database. This is because α- and β-anomers reach equilibrium through mutarotation; thus, the relative abundance of α- and β-anomers remains the same. (3) Comparison of the MS^2^ spectrum at the corresponding dextran indices with those in the database. Notably, the intensity in the chromatogram is the total ionic fragment intensity of MS^2^; thus, the MS^2^ mass spectra are necessary to be measured. The MS^2^ CID spectra usually have a very good signal-to-noise ratio and can be used for the differentiation of some isomers. Most peaks in chromatograms can be identified using these three criteria. Using dextran indices instead of the retention times is particularly useful if the change of retention time is due to the PGC columns from different manufacturers. If two or more than two isomers have similar dextran indices and MS^2^ spectra, the following criteria are used for differentiation, i.e., (4) comparison of two MS^3^ mass spectra at the corresponding dextran indices with those in the database. If the dextran indices, MS^2^, and MS^3^ spectra are similar between isomers, additional criteria are used, i.e., (5) comparison of MS^4^ spectra at the corresponding dextran indices with those in the database. The CID sequences for the MS^3^ and MS^4^ spectra are illustrated in the database.

#### Bovine Whey Proteins

**Figure 5:**
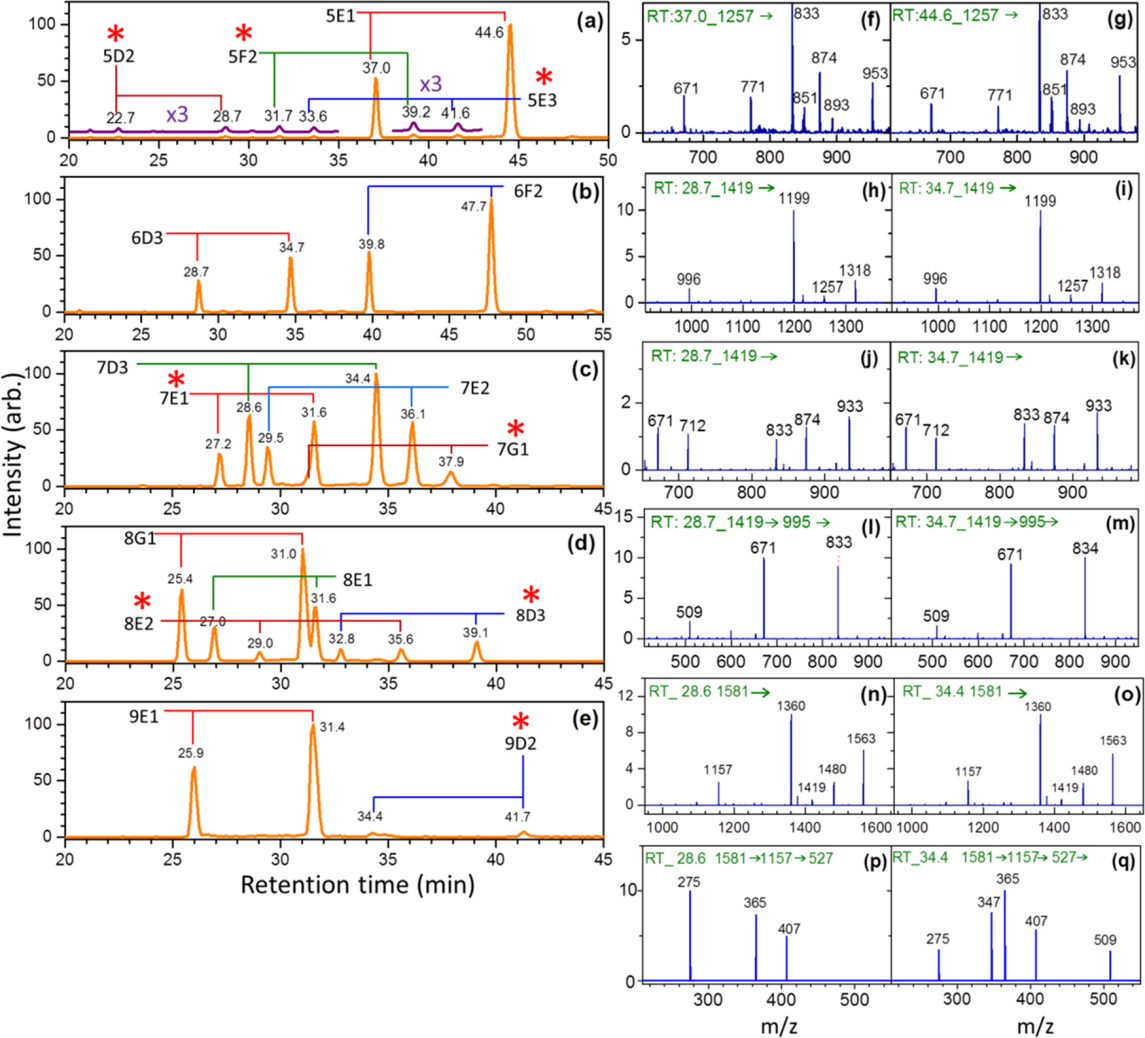
Chromatograms (a–e) and CID spectra (f–q) of Hex*_n_*GlcNAc_2_ (*n* = 5–9) extracted from bovine whey proteins (released by an ammonia-catalyzed reaction). Structural assignment was based on three criteria: (1) The retention time of the ions with the selected *m*/*z* values, (2) CID MS*^n^* spectra, and (3) the relative intensity of two anomers of the same isomer. Isomers denoted by red stars represent the isomers that have not been reported in previous studies of bovine whey proteins.

The structures of *N*-glycans in bovine whey proteins have been studied by several research groups.^7,40−42^ In this study, we applied the aforementioned database to determine the structures of high-mannose *N*-glycans in bovine whey proteins. Figure 5a–e provides the chromatogram of ions *m*/*z* 1257, 1419, 1581, 1743, and 1905, representing sodium ion adducts of Hex*_n_*GlcNAc_2_ (*n* = 5–9) extracted from bovine whey proteins.

Here, we used the *N*-glycans extracted from bovine whey proteins as examples to demonstrate how to use the database to identify the structures of *N*-glycans for the peaks in chromatogram. In the structure identification of Man_5_GlcNAc_2_ in Figure 5a, we noted that there are two peaks with large intensities. Notably, the intensities of two peaks in the chromatogram of the same isomer are always very similar, as illustrated in the database. Thus, the two peaks (*t* = 37.0 and 44.6 min) with large intensities in Figure 5a must belong to the same isomer. There are two isomers with retention times close to *t* = 37.0 and 44.6 min. One is isomer 5E2, the other is isomer 5E1. The MS^2^ spectra at *t* = 37.0 and 44.6 min, illustrated in Figure 5f,g, show that the intensities of ions *m*/*z* 712 and 933 are much lower than that of ions *m*/*z* 671 and 771. These MS^2^ spectra suggest the isomer at *t* = 37.0 and 44.6 min is 5E1 by comparing to the Man_5_GlcNAc_2_ database in AppendixSection. The next step is the assignment of the peaks with small intensities located at *t* = 22.7, 28.7, 31,7, 33.6, 39.2, and 41.6 min in Figure 5a. There is only one isomer, namely, 5E3, with retention time near *t* = 41.6 min. The retention times of isomer 5E3 are *t* = 41.6 and 33.7 min according to the database. Thus, the peak at *t* = 33.6 min in Figure 5a, with intensity similar to the peak at *t* = 41.6 min in Figure 5a, can be assigned to 5E3. The analogous procedure for the structure assignments can be made for the other four peaks. There is only one isomer, namely, 5D2, with retention time near *t* = 22.7 min. Compared to the database of isomer 5D2 (*t* = 22.7 and 28.8 min), the peak at *t* = 28.7 min in Figure 5a, with intensity similar to the peak at *t* = 22.7 min in Figure 5a, is assigned to 5D2. Finally, there is only one isomer, namely, 5F2, with retention time near *t* = 39.2 min. Compared to the database of isomer 5F2 (*t* = 30.8 and 38.9 min), the peak at *t* = 31.7 min in Figure 5a, with intensity similar to the peak at *t* = 39.2 min in Figure 5a, is assigned to 5F2.

There is only one isomer, namely, 6F2, with retention time near the peak *t* = 47.7 min in Figure 5b. According to the database (6F2 are *t* = 47.5 and 39.2 min, illustrated in Appendix Section), the peak at *t* = 39.8 min in Figure 5b, with intensity a little smaller than that of the peak at *t* = 47.7 min in Figure 5b, is assigned to isomer 6F2. There are four candidates, namely, 6H1, 6F1, 6D3, and 6E2 for the peaks at *t* = 28.7 and 34.7 min in Figure 5b. Compared to the MS^2^ database of Man_6_GlcNAc_2_ in Appendix Section, the MS^2^ spectrum [Figure 5h,i] of the peaks at *t* = 28.7 and 34.7 min shows that the intensity of ion *m*/*z* 1257 is much smaller than that of ion *m*/*z* 1318, suggesting it is not 6H1. The high intensities of ions *m*/*z* 712 and 933 and the low intensity of ion *m*/*z* 771, shown in Figure 5j,k, exclude the possibility of isomer 6F1. Comparing the MS^3^ spectrum of the peaks at *t* = 28.7 and 34.7 min [Figure 5l,m] to the MS^3^ spectrum in database (in Appendix Section) suggests it is isomer 6D3.

In Figure 5c, there is a peak at retention time *t* = 37.9 min. Because only the retention time of isomer 7G1 is near *t* = 37.9 min, it is assigned to 7G1. According to the database, the other peak of 7G1 is located near *t* = 31.2 min. There is a peak at *t* = 31.6 min, but the intensity is too large compared to that of the peak at *t* = 37.9 min in Figure 5c. It is likely due to the overlap of the other isomer at *t* = 31.6 min. The peak at *t* = 27.2 min is assigned to isomer 7E1 since isomer 7E1 is the only isomer with retention time near 27.2 min. The other peak of isomer 7E1 is located at *t* = 31.7 min, according to the database. Thus, the high intensity at *t* = 31.6 min in Figure 5c can be attributed to the overlap of isomer 7G1 at *t* = 31.2 min and isomer 7E1 at *t* = 31.7 min in the database. The peak at *t* = 36.1 min is assigned to 7E2 because only isomer 7E2 has a peak close to *t* = 36.1 min. According to the database, the retention times of isomer 7E2 are *t* = 36.1 and 29.5 min. Thus, the peak at *t* = 29.5 min in Figure 5c, with intensity a little smaller than that of the peak at *t* = 36.1 min in Figure 5c, is assigned to isomer 7E2. There are three candidates, namely, 7F1, 7D3, and 7D1, for the peaks located at *t* = 34.4 and 28.6 min in Figure 5c. The MS^2^ spectrum of the peaks at *t* = 34.4 and 28.6 min through the CID sequence 1581→ [Figure 5n,o] shows that the intensity of ion *m*/*z* 1581 is much larger than that of ion *m*/*z* 1419, indicating it is not 7F1. Meanwhile, the MS^4^ spectrum through the CID sequence 1581 → 1157 → 527→ [Figure 5p,o,q] shows a large intensity of ion *m*/*z* 275, and no ions *m*/*z* 437 and 467 were found, suggesting only isomer 7D3 existed.

The peaks at *t* = 25.4 and 27.0 min in Figure 5d are assigned to isomers 8G1 and 8E1, respectively, because only the isomers 8G1 and 8E1 have similar retention times in the database (8G1: *t* = 25.3 min; 8E1: t = 26.7 min). The peaks at *t* = 31.0 and 31.6 min are assigned to the other peaks of isomers 8G1 and 8E1, respectively, according to the retention times and relative intensity of 8G1 and 8E1 in the database (8G1 *t* = 30.8 min; 8E1 *t* = 31.4 min). The peaks at *t* = 39.1 and 32.5 min are assigned to 8D3, and the peaks at 35.6 and 29.0 min are assigned to 8E2 based on the retention times of database (8D3 *t* = 32.8 and 39.2 min; 8E2 *t* = 29.1 and 35.5 min).

The two peaks with large intensity at *t* = 25.9 and 31.4 min in Figure 5e must belong to one isomer, while the two peaks with small intensity at *t* = 34.4 and 41.7 min in Figure 5e belong to the other isomer. Compared to the retention times in the database (9E1: *t* = 25.7 and 31.5 min; 9D2: *t* = 34.2 and 41.2 min), they are assigned to 9E1 and 9D2, respectively.

Many *N*-glycans that have not been found in bovine whey proteins before were identified in this study. Among these newly discovered high-mannose *N*-glycans, Man_5_GlcNAc_2_ isomers are noteworthy. The conventional multicellular eukaryote *N*-glycan biosynthesis suggests that high-mannose *N*-glycans are generated by conserved biosynthetic pathways. After the oligosaccharide of Glc_3_Man_9_GlcNAc_2_ is transferred from lipid to proteins, glucoses and mannose are removed sequentially by various glucosidases and α-1,2-mannosidases. Through this biosynthetic pathway, four Man_7_GlcNAc_2_ isomers, three Man_6_GlcNAc_2_ isomers, and one Man_5_GlcNAc_2_ isomer are generated. However, a recent study reported the presence of isomers beyond those generated through the current biosynthetic pathways in various biological samples.^36^ In the present study, we found three Man_5_GlcNAc_2_ isomers that were not expected to be generated according to the conventional biosynthetic pathway, although their abundance was low.

#### Soybean (*Glycine max*)

**Figure 6:**
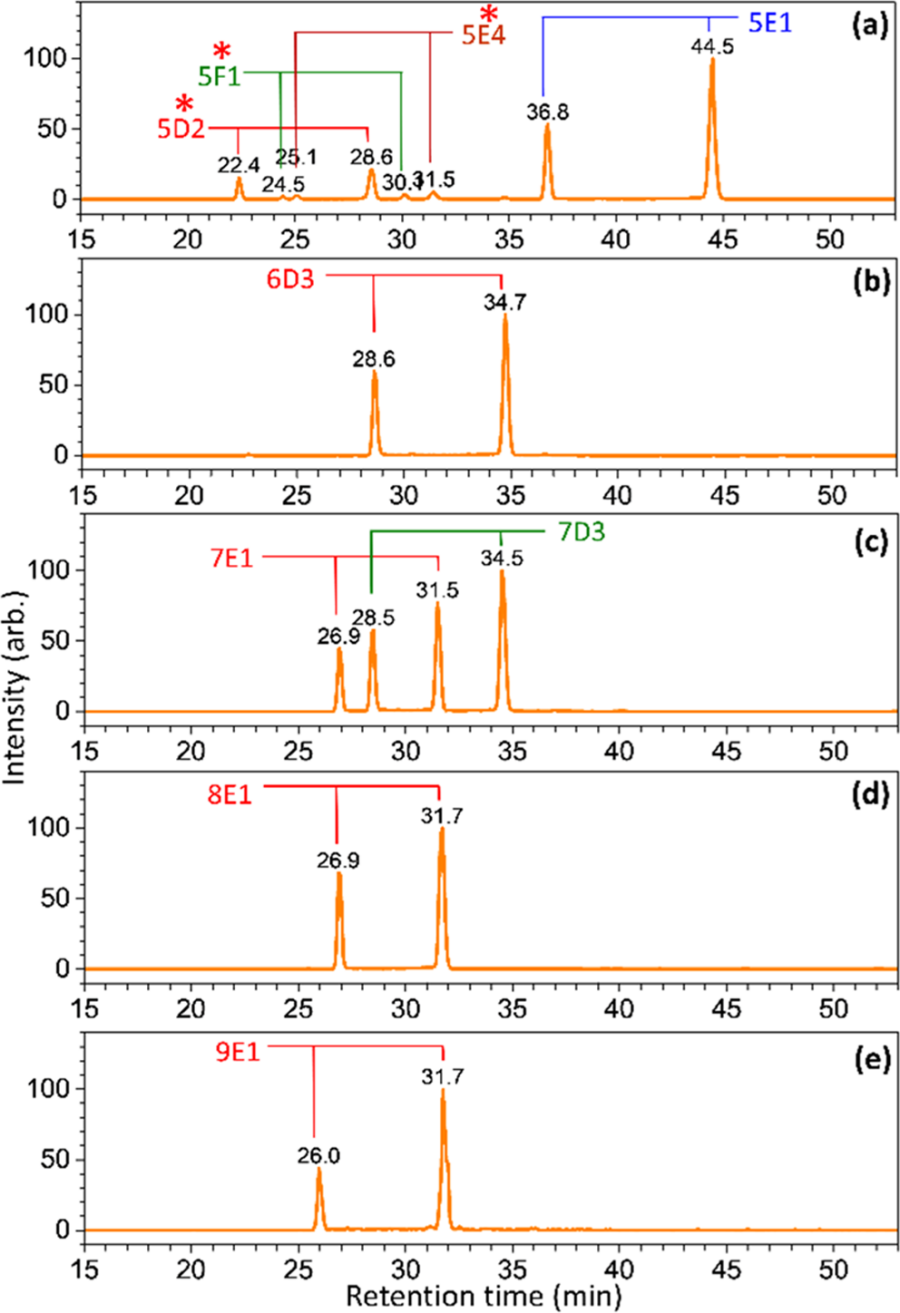
Chromatograms of ions with *m*/*z* values of (a) 1257, (b) 1419, (c) 1581, (d) 1743, and (e) 1905, representing the sodium ion adducts of Hex*_n_*GlcNAc_2_ (*n* = 5–9) extracted from soybean proteins (released by the enzyme PNGase F). Isomers denoted by red stars represent the isomers that have not been found in previous studies of soybean proteins.

The structures of *N*-glycans of soybean have been investigated for more than two decades.^43−47^ In a recent study, soybean proteins were used as the source for the large-scale preparation of high-mannose and paucimannose *N*-glycans.^48^Figure 6 presents the chromatograms of ions *m*/*z* 1257, 1419, 1581, 1743, and 1905, representing the sodium ion adducts of Hex_n_GlcNAc_2_ (*n* = 5–9) extracted from soybean proteins. This study discovered three Man_5_GlcNAc_2_ isomers that have not been reported before and that were not expected to be generated according to the conventional biosynthetic pathways. The abundances of these Man_5_GlcNAc_2_ isomers were not very low compared with that of the canonical Man_5_GlcNAc_2_ isomer 5E1. Among these three isomers, only one is the same as the Man_5_GlcNAc_2_ isomers found in bovine whey proteins.

#### Human Mammary Epithelial Cells and Breast Carcinoma

**Figure 7:**
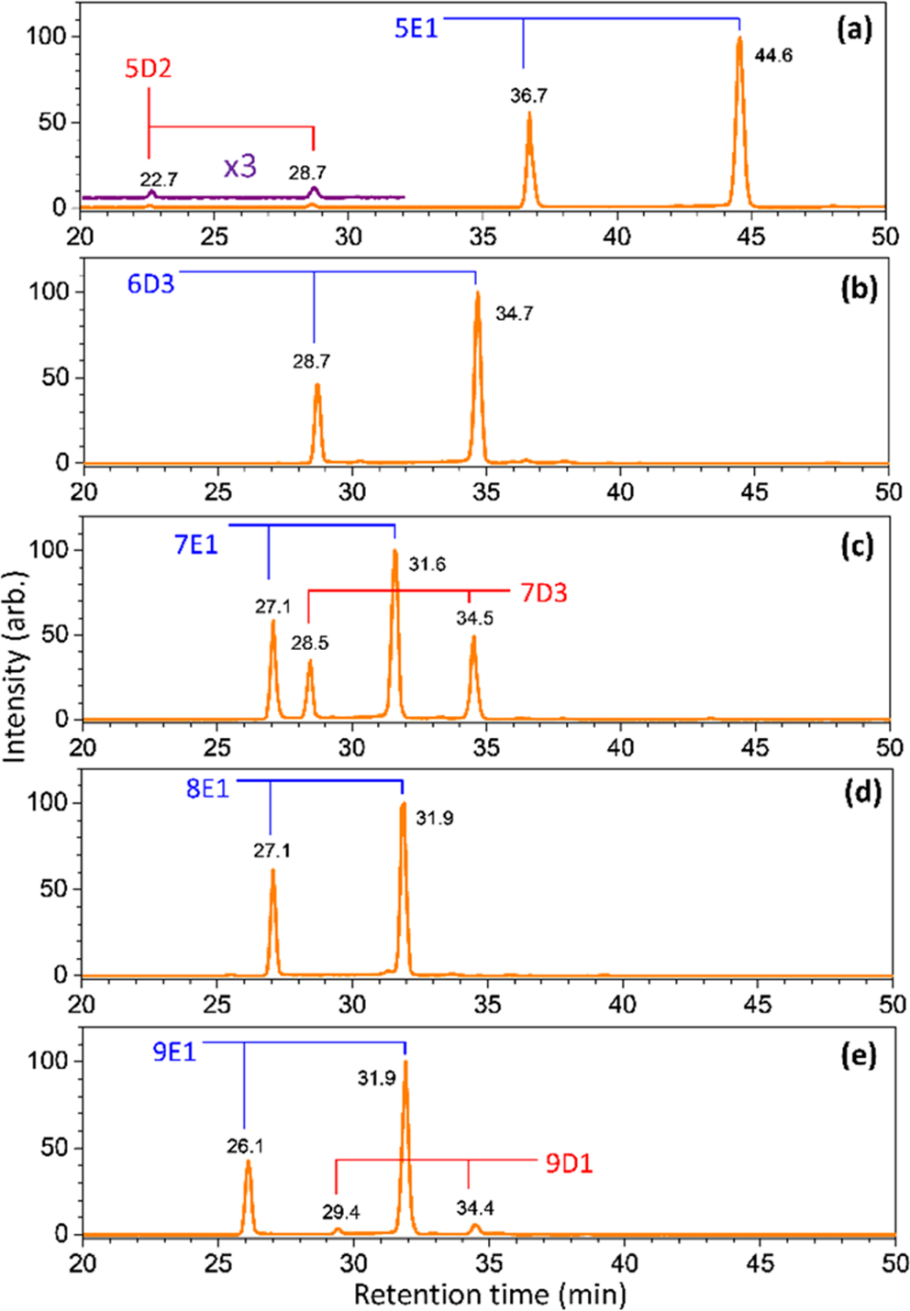
Chromatograms of ions with *m*/*z* values of (a) 1257, (b) 1419, (c) 1581, (d) 1743, and (e) 1905, representing the sodium ion adducts of Hex_n_HexNAc_2_ (*n* = 5–9) extracted from SKBR-3 (HER2-overexpressing subtype human breast carcinoma) released by the enzyme PNGase F.

Unusual changes in glycans have been found to be associated with tumorigenesis, tumor progression, and metastasis.^49−51^ High-mannose *N*-glycans are elevated during breast cancer progression; however, the detailed structures of these high-mannose *N*-glycans are unknown.^52−54^ In this study, we investigated the following human cell lines: M10 (human mammary epithelial cells), MCF-7 (luminal A subtype human breast carcinoma), SKBR-3 (HER2-overexpressing subtype human breast carcinoma), MDA-MB-231 (basal-like subtype human breast carcinoma), and BT-549 (triple-negative human breast carcinoma). The chromatograms of high-mannose *N*-glycans extracted from the SKBR-3 cell line are illustrated in Figure 7, the chromatograms of the other cell lines are illustrated in Section(F) in the Supporting Information. We found similar high-mannose *N*-glycan isomer distribution profiles for all of the cell lines, except two minor differences. First, there are three isomers of Man_5_GlcNAc_2_, 5D2, 5E1, and 5E4, in human mammary epithelial cells, but there are only two isomers of Man_5_GlcNAc_2_, 5D2 and 5E1 in cancer cell lines. Second, the intensity of 7D3 is smaller than that of 7E1 for all cell lines, except for BT-549 which the intensities of 7D3 and 7E1 are similar.

## Conclusions

In this study, we constructed a database of high-mannose and paucimannose *N*-glycans; this database included the retention time in chromatograms and CID MS*^n^* mass spectra. These *N*-glycans included the isomers by breaking of arbitrary numbers of glycosidic bonds at arbitrary positions of mannoses from Man_9_GlcNAc_2_. In addition, a part of the retention time and CID spectra of GlcMan_6–9_GlcNAc_2_ was included. The database enables the rapid assignment of structures for paucimannose and high-mannose *N*-glycans, and it is particularly useful for the discovery of the *N*-glycans not in the conventional *N*-glycan standards. Applications of the database to the structural assignment of the *N*-glycans extracted from various biological samples were demonstrated, and many high-mannose *N*-glycans that are not expected to be generated according to the conventional biosynthetic pathway were found.

## Appendix

### Database of Hex*_n_*GlcNAc_2_ (*n* = 4–10)

#### Man_4_GlcNAc_2_

**Figure 8:**
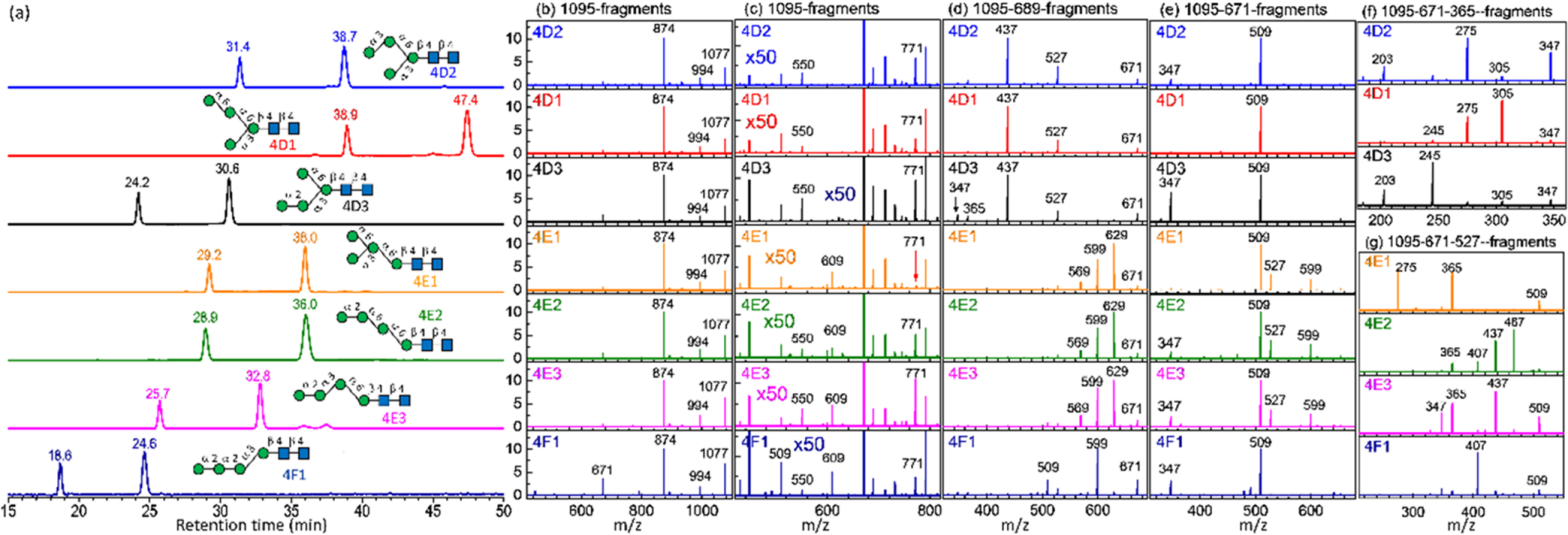
Database of Man_4_GlcNAc_2_. (a) Chromatogram. CID spectra of sodium ion adducts based on the CID sequence of (b) and (c) 1095 → fragments, (d) 1095 → 689 → fragments, (e) 1095 → 671 → fragments, (f) 1095 → 671 → 365 → fragments, (g) 1095 → 671 → 527 → fragments. All *y*-axes represent intensity in arbitrary units. All isomers are commercial products.

There are seven isomers of Man_4_GlcNAc_2_. All isomers were purchased from Omicron Biochemicals, Inc. The chromatograms and CID spectra are illustrated in Figure 8a,b–g, respectively. The LODES for Man_4_GlcNAc_2_ isomer differentiation is illustrated in Section(B) in the Supporting Information. The retention times of these isomers are very different, except for isomers 4E1 and 4E2. The differentiation of isomers 4E1 and 4E2 relies on the mass spectra.

The major fragments in MS^2^ spectra of all isomers were similar, except for that of isomer 4F1 [Figure 8b]; the intensity of ion *m*/*z* 671 was higher than that of ion *m*/*z* 994. The fragments of low intensities in MS^2^ are illustrated in Figure 8c. Fragment *m*/*z* 771 results from the loss of two mannoses, which can be produced easily from all isomers by breaking one glycosidic bond, except isomer 4E1 which requires the cleavage of two glycosidic bonds. Thus, the intensity of fragment *m*/*z* 771 is low for isomer 4E1, and it can be used to differentiate isomer 4E1 from the other isomers. Fragment *m*/*z* 609 results from the loss of two mannoses, which can be generated by breaking one glycosidic bond from isomers 4E1, 4E2, 4E3, and 4F1. Thus, the intensities of fragment *m*/*z* 609 of isomers 4E1, 4E2, 4E3, and 4F1 are larger than the other isomers, and it can be used to differentiate isomers 4E1, 4E2, 4E3, and 4F1 from the other isomers. Fragment *m*/*z* 550 results from the loss of two mannoses and one GlcNAc, which can be produced from all isomers by breaking two glycosidic bonds, except isomer 4E1 which requires the cleavage of three glycosidic bonds. Thus, the intensity of fragment *m*/*z* 550 is very low, and it can be used to differentiate isomer 4E1 from the other isomers.

According to the CID spectra of the sequence, 1095 → 689 → fragments, through C_*n*–2_ ion (*m*/*z* 689), isomers were classified into three groups, namely, D, E, and F, by comparing the intensities of fragment ions with *m*/*z* values of 629, 599, 569, and 437. Isomers in the D group were differentiated based on the CID spectra of 1095 → 671 → 365 → fragments by comparing the intensities of ions with *m*/*z* values of 245, 275, and 305. Isomers in the E group were distinguished based on the CID spectra of 1095 → 671 → 527 → fragments by comparing the intensities of ions with *m*/*z* values of 467, 437, 407, and 275. All of the fragment ions produced by the cross-ring dissociation of C ions, including C_*n*–2_ ion (*m*/*z* 689) based on the CID sequence of 1095 → 689 → fragments, C_2_ (*m*/*z* 365) ion based on the CID sequence of 1095 → 671 → 365 → fragments, and C_3_ (*m*/*z* 527) ion based on the CID sequence of 1095 → 671 → 527 → fragments, follow the retro-aldol reaction closely. For example, in the CID sequence of 1095 → 689 → fragments, a high intensity of ion *m*/*z* 437 and near-zero intensity of ions *m*/*z* 629, 599, and 569 were found for isomers 4D1, 4D2, and 4D3; high intensities of ions *m*/*z* 629, 599, and 569 and near-zero intensity of ion *m*/*z* 437 were found for isomers 4E1, 4E2, 4E3; and high intensity of ion *m*/*z* 599 and near-zero intensity of ions *m*/*z* 437, 569, 599, and 629 were found for isomer 4F1.

Isomers 4E1 and 4E2 have similar retention times. Differentiation of these two isomers can be made using the following methods. First, these two isomers could be distinguished using the spectra based on the CID sequence of 1095 → 671 → fragments [Figure 8e]: the intensity of the ion with the *m*/*z* value of 347 was different. The second method is the MS^4^ spectrum through the CID sequence, 1095 → 671 → 527 → fragments [Figure 8g], as discussed in the previous paragraph. The third method is the shortcut CID sequence, 1095 → 527 → fragments. The shortcut CID sequence reduces the number in the multistage CID sequence, changing from 1095 → 671 → 527 → fragments to 1095 → 527 → fragments, thus enhancing the signal-to-noise ratio. However, this shortcut CID sequence may produce fragments *m*/*z* 467 and 437 from isomer 4E1 or fragment *m*/*z* 275 from isomer 4E2 due to secondary dissociation, but the intensities of these fragments are small and isomers 4E1 and 4E2 can be differentiated. The fourth method is the fragments *m*/*z* 771 and 550 in MS^2^ spectrum [Figure 8c]. Although the intensities of fragments *m*/*z* 771 and 550 are small, the signal-to-noise ratio is good because only one stage of CID was used in MS^2^. The fourth method is particularly useful when the concentration of the sample in HPLC is low.

#### Hex_5_GlcNAc_2_

**Figure 9:**
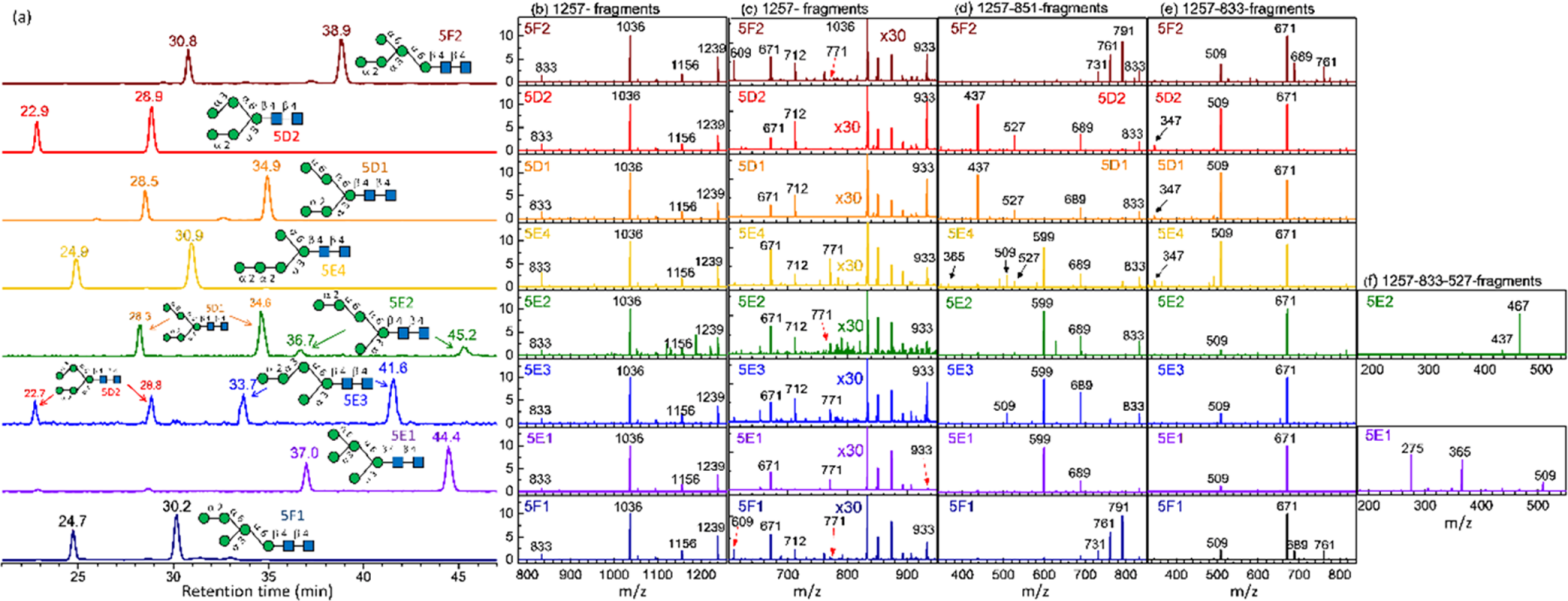
Database of Man_5_GlcNAc_2_. (a) Chromatogram. CID spectra of sodium ion adducts based on the CID sequence of (b) and (c) 1257 → fragments, (d) 1257 → 851 → fragments, (e) 1257 → 833 → fragments, and (f) 1257 → 833 → 527 → fragments. All *y-*axes represent intensity in arbitrary units. 5D1 and 5D2 were commercial products; 5E1 and 5F1 were extracted from black bean; 5E4 and 5F2 was generated from degradation of 6E2 and 6G1 by enzyme, respectively; 5E2 and 5E3 were generated from degradation of 7D1 and 7D2, respectively.

There are eight isomers of Man_5_GlcNAc_2_. Isomers 5E1 and 5F1 were extracted from black bean, and isomers 5D1 and 5D2 were purchased from Omicron Biochemicals, Inc. Isomer 5E4 was generated from the degradation of the Man_6_GlcNAc_2_ isomer 6E2; this reaction was catalyzed by α1–6 mannosidase. Isomer 5F2 was produced from the degradation of the Man_6_GlcNAc_2_ isomers 6G1, which was catalyzed by α-mannosidase from *C. ensiformis* (Jack bean); isomers 5E2 and 5E3 were generated from the degradation of the Man_7_GlcNAc_2_ isomers 7D1 and 7D2, respectively, which was catalyzed by α-mannosidase from *C. ensiformis*. The degradation of large *N*-glycans may generate more than one Man_5_GlcNAc_2_ isomer. In this study, we had data on the retention time and CID spectra of all of the potential isomers enzymatically generated, except for the isomer of interest. Therefore, we could accurately assign the structures of the isomers generated by enzymes. For example, the degradation of the Man_6_GlcNAc_2_ isomer 6G1 by α-mannosidase from *C. ensiformis* generated two Man_5_GlcNAc_2_ isomers, namely, 5F1 and 5F2. Because we had the data of isomer 5F1 extracted from black bean, we assigned the enzyme-generated isomer, which had a retention time different from that of isomer 5F1, to isomer 5F2. The CID spectra for the structural determination of these isomers have been reported in our previous study.^36^ The chromatogram and CID spectra are illustrated in Figure 9.

According to the retro-aldol reaction, LODES predicted that the spectra based on the CID sequence of 1257 → 851 → fragments through C_*n*–2_ ion (*m*/*z* 851) were different for groups D, E, F, and G, as illustrated in Figure 9d. A high intensity of ions with *m*/*z* values of 791, 761, and 731 and near-zero intensity of ions with *m*/*z* values of 599 and 437 were found for isomers in group F; a high intensity of ions with an *m*/*z* value of 599 and near-zero intensity of ions with *m*/*z* values of 791, 761, 731, and 437 were found for isomers in group E; a high intensity of ions with an *m*/*z* value of 437 and near-zero intensity of ions with *m*/*z* values of 791, 761, 731, and 599 were found for isomers in the D group.

Starting from Man_5_GlcNAc_2_ to larger high-mannose *N*-glycans, the database included one MS^2^ and two MS^3^ (through C_*n*–2_ and B_*n*–2_ ions) spectra. These spectra obtained through HPLC/ESI/MS real-time online measurement exhibited a high signal-to-noise ratio. However, these spectra are insufficient for determining *N*-glycan structures. The complete CID spectra for structural determination are presented in a previous report^36^ or in Section(C) in the Supporting Information in the present study. Although isomer structural identification cannot be made solely using MS^2^ and MS^3^ spectra, most structures are assigned explicitly by using the retention time in chromatograms and the MS^2^ and MS^3^ CID spectra in the database.

The retention time of isomers 5E1 and 5E2 was similar. These two isomers can be distinguished using the following methods. The MS^4^ spectra through the CID sequence 1257 → 833 → 527 → fragments, as shown in Figure 9f, can be used to distinguish between these two isomers if the concentrations of these isomers in solution were not too low. Alternatively, the shortcut CID sequence, 1257 → 527 → fragments, can be used. In this shortcut sequence, the CID spectra are similar to that in Figure 9f with additional small intensities of *m*/*z* 275 in 5E2 and *m*/*z* 467 in 5E1 due to the interference by secondary dissociation. Another method is the fragments *m*/*z* 933 and 712 in MS^2^ spectrum [Figure 9c]. Fragment *m*/*z* 933 results from the loss of two mannoses in CID which can be produced from isomer 5E2 by breaking one glycosidic bond, but it requires the cleavage of two glycosidic bonds for isomer 5E1. Thus, the fragment *m*/*z* 933 in the CID MS^2^ spectrum of isomer 5E2 must be much larger than that of isomer 5E1. Fragment *m*/*z* 712 results from the loss of two mannoses and one GlcNAc which can be generated by breaking two glycosidic bonds from isomer 5E2 but it requires the cleavage of three bonds for isomer 5E1. Thus, the intensity of fragment *m*/*z* 712 in the CID MS^2^ spectrum of isomer 5E2 must be much larger than that of isomer 5E1.

The retention time of isomers 5E4 and 5F1 was not very different. These two isomers can be distinguished using the MS^3^ spectra [Figure 9d,e]. Alternatively, they can be distinguished using the fragments 609 and 771 in MS^2^ spectrum. Fragment *m*/*z* 609 results from the elimination of four mannoses which requires breaking one glycosidic bond for isomer 5F1 but two bonds for isomer 5E4. Thus, the fragment *m*/*z* 609 in the CID MS^2^ spectrum of isomer 5F1 is much larger than that of isomer 5E4. Fragment *m*/*z* 771 results from the loss of three mannoses which requires breaking one glycosidic bond for isomer 5E4 but two bonds for isomer 5F1. Thus, the fragment *m*/*z* 771 in the CID MS^2^ spectrum of isomer 5E4 is much larger than that of isomer 5F1.

#### Hex_6_GlcNAc_2_

**Figure 10:**
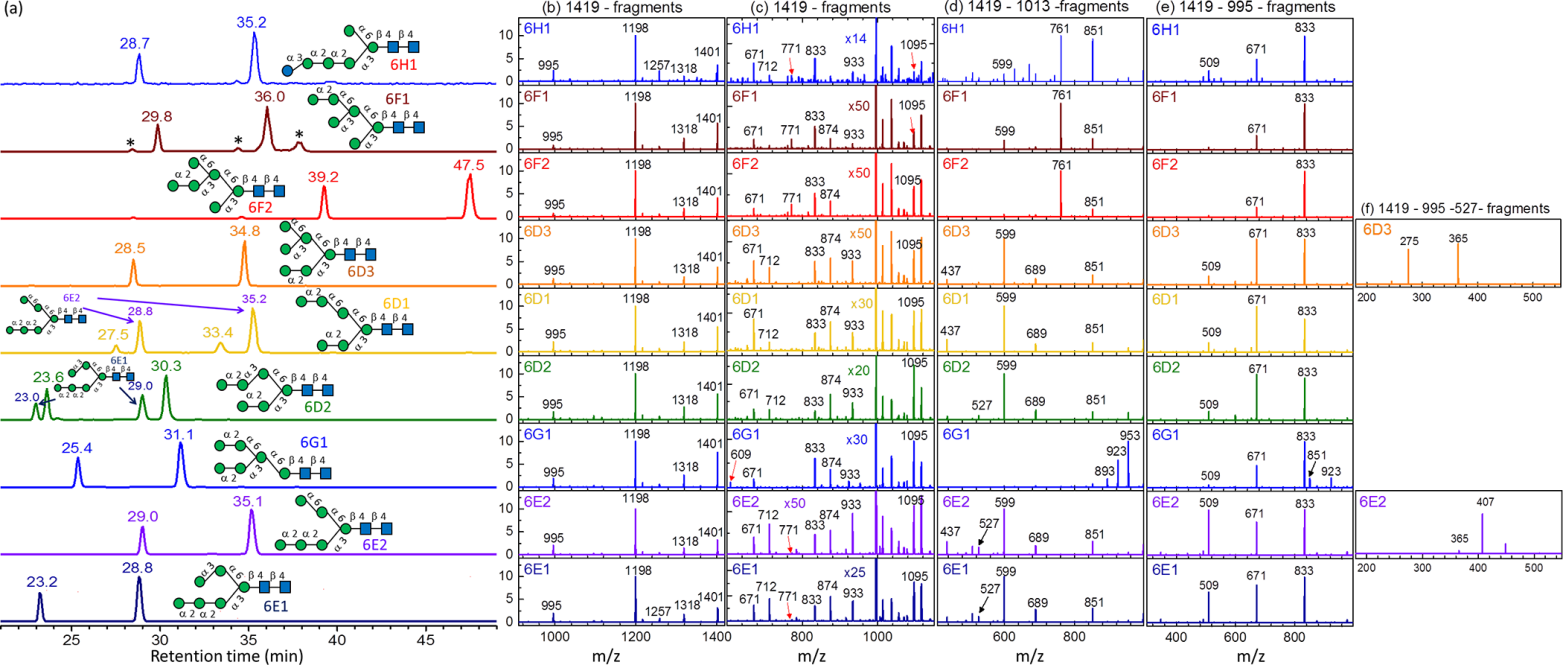
Database of Man_6_GlcNAc_2_ and GlcMan_5_GlcNAc_2_. (a) Chromatogram. CID spectra of sodium ion adducts based on the CID sequence of (b) and (c) 1419 → fragments, (d) 1419 → 1013 → fragments, (e) 1419 → 995 → fragments, and (f) 1419 → 995 → fragments. All *y-*axes represent intensity in arbitrary units. 6E1, 6E2, and 6G1 were commercial products; 6D3 and 6F2 were extracted from bovine whey proteins; 6F1 was extracted from black bean; 6D1 and 6D2 were generated from degradation of 7D1 and 7D2 by enzyme, respectively. 6H1 was produced using three methods: degradation of 8D3 by enzyme, degradation of 9D1 or 9D2 by enzyme, and extraction from hen egg IgY.

There are eight isomers of Man_6_GlcNAc_2_. Isomers 6E1, 6E2, and 6G1 were purchased from Omicron Biochemicals, Inc.; isomers 6D3 and 6F2 were extracted from bovine whey proteins; and isomer 6F1 was extracted from black bean. A part of the CID spectra for the structural determination of these isomers has been presented in our previous study,^36^ and the rest of the CID spectra are illustrated in Section(C) in the Supporting Information. Isomers 6D1 and 6D2 were generated from the degradation of the Man_7_GlcNAc_2_ isomers 7D1 and 7D2, respectively, in a reaction catalyzed by α-mannosidase of *C. ensiformis*. In addition, the GlcMan_5_GlcNAc_2_ isomer 6H1 was included in the database. It was produced using three methods: the degradation of GlcMan_7_GlcNAc_2_ isomer 8D3 by α-mannosidase from *C. ensiformis*, the degradation of the GlcMan_8_GlcNAc_2_ isomers 9D1 or 9D2 by α-mannosidase from *C. ensiformis*, and the extraction of the isomer from hen egg IgY. The chromatograms and CID spectra of the database are presented in Figure 10.

According to the retro-aldol reaction, LODES predicted a high intensity of ions *m*/*z* 953, 923, and 893 for isomer 6G1; a high intensity of ion *m*/*z* 761 for isomers in groups F and H; and a high intensity of ion *m*/*z* 599 for isomers in groups D and E based on the CID spectra of sequence 1419 → 1013 → fragments through C_*n*–2_ ion [Figure 10d].

One interesting observation is the easy elimination of Glc from isomer 6H1. The intensity of fragment ion *m*/*z* 1257 (loss of a hexose) of isomer 6H1 based on CID sequence of 1419 → fragments and the intensity of fragment ion *m*/*z* 851 (loss of a hexose) of isomer 6H1 based on CID sequence 1419 → 1013 → fragments were higher than those of the other isomers [Figure 10b,d]. This is because, among all isomers, glucose in isomer 6H1 is the only monosaccharide with O1 and O2 atoms in the cis configuration. In our previous studies, we demonstrated that O1 and O2 in the cis configuration promote glycosidic bond cleavage.^26,27^ The high intensities of the ions with *m*/*z* values of 1257 and 851 in MS^2^ and MS^3^, respectively are useful for the identification of isomer 6H1.

The retention times of isomers 6D3, 6E2, 6F1, and 6H1 are not very different. Isomer 6H1 can be distinguished from the others by the relatively large intensity of *m*/*z* 1257 in MS^2^. Isomers 6F1 and 6H1 can be distinguished from 6D3 and 6E2 using fragment *m*/*z* 771 in MS^2^ or fragment *m*/*z* 761 in MS^3^ through the CID sequence of 1419 → 1013 → fragments. Isomers 6D3, 6E2, and 6F1 can be distinguished from each other by the relative intensities of fragments *m*/*z* 509, 671, and 833 through the CID sequence of 1419 → 995 → fragments. Isomers 6D3 and 6E2 can be differentiated by the fragments *m*/*z* 275 and 407 based on the CID sequence 1419 → 995 → 527 → fragments, or the shortcut CID sequence 1419 → 527 → fragments.

#### Hex_7_GlcNAc_2_

**Figure 11:**
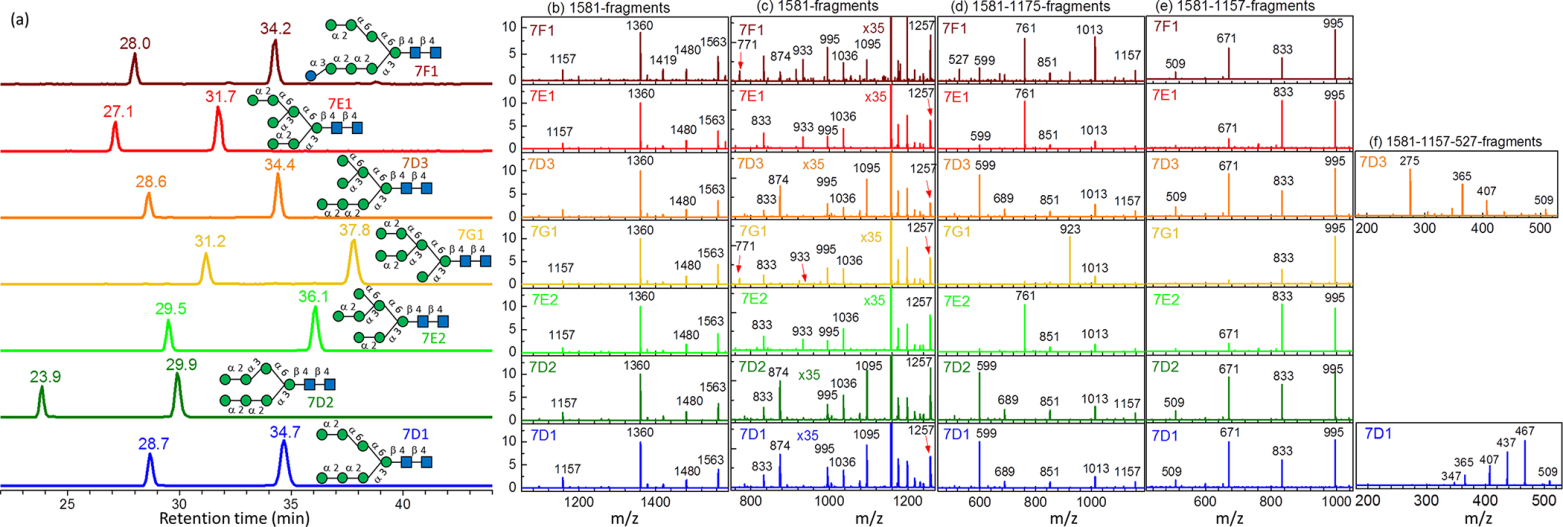
Database of Man_7_GlcNAc_2_ and GlcMan_6_GlcNAc_2_. (a) Chromatogram. CID spectra of sodium ion adducts based on the CID sequence of (b) and (c) 1581 → fragments, (d) 1581 → 1175 → fragments, (e) 1581 → 1157 → fragments, and (f) 1581 → 1157 → 527 → fragments. All *y-*axes represent intensity in arbitrary units. 7D1 and 7D2 were commercial products; 7E1, 7E2, 7D3, and 7G1 were extracted from bovine lactoferrin; 7F1 was extracted from hen egg IgY or produced by degradation of 8D3 by enzyme or degradation of 9D1 and 9D2 by enzyme.

There are seven isomers of Man_7_GlcNAc_2_. Among these seven isomers, isomers 7D1 and 7D2 were purchased from Omicron Biochemicals, Inc., and isomers 7E1, 7E2, 7D3, and 7G1 were extracted from bovine lactoferrin. One isomer of GlcMan_7_GlcNAc_2_, 7F1, was extracted from hen egg IgY and was cross-checked through the degradation of the GlcMan_7_GlcNAc_2_ isomer 8D3 by α-mannosidase from *C. ensiformis* and the degradation of the GlcMan_8_GlcNAc_2_ isomers 9D1 and 9D2 by α-mannosidase from *C. ensiformis*. A part of the CID spectra for structural determination has been provided in a previous report,^36^ and the rest of the CID spectra are presented in (C) CID spectra in the Supporting Information. The chromatograms and the CID spectra of the database are illustrated in Figure 11.

The relative intensities of ions *m*/*z* 599, 761, and 923 in the spectra based on the CID sequence of 1581 → 1175 → fragments through C_*n*–2_ ion (*m*/*z* 1175) followed the retro-aldol reaction closely [Figure 11d]. The easy elimination of glucose was also observed for isomer 7F1; a high intensity was found for fragment ions *m*/*z* 1419 and 1013 through the CID sequences of 1581 → fragments [Figure 11b] and 1581 → 1175 → fragments [Figure 11d], respectively.

Among these isomers, the retention times of isomers 7D1, 7D3, and 7F1 were similar. Isomer 7F1 could be distinguished from the others based on the fragment *m*/*z* 1419 in the MS^2^ spectrum, or *m*/*z* 761 and 1013 in the MS^3^ spectrum through the CID sequence of 1581 → 1175 → fragments. Isomers 7D1 and 7D3 could be distinguished using the MS^4^ CID spectrum [1581 → 1157 → 527 → fragments; Figure 11f] or based on the shortcut CID sequence of 1581 → 527 → fragments; large intensities of fragments *m*/*z* 467 and 437 were found in isomer 7D1, and large intensity of fragment *m*/*z* 275 was found in isomer 7D3.

#### Hex_8_GlcNAc_2_

**Figure 12:**
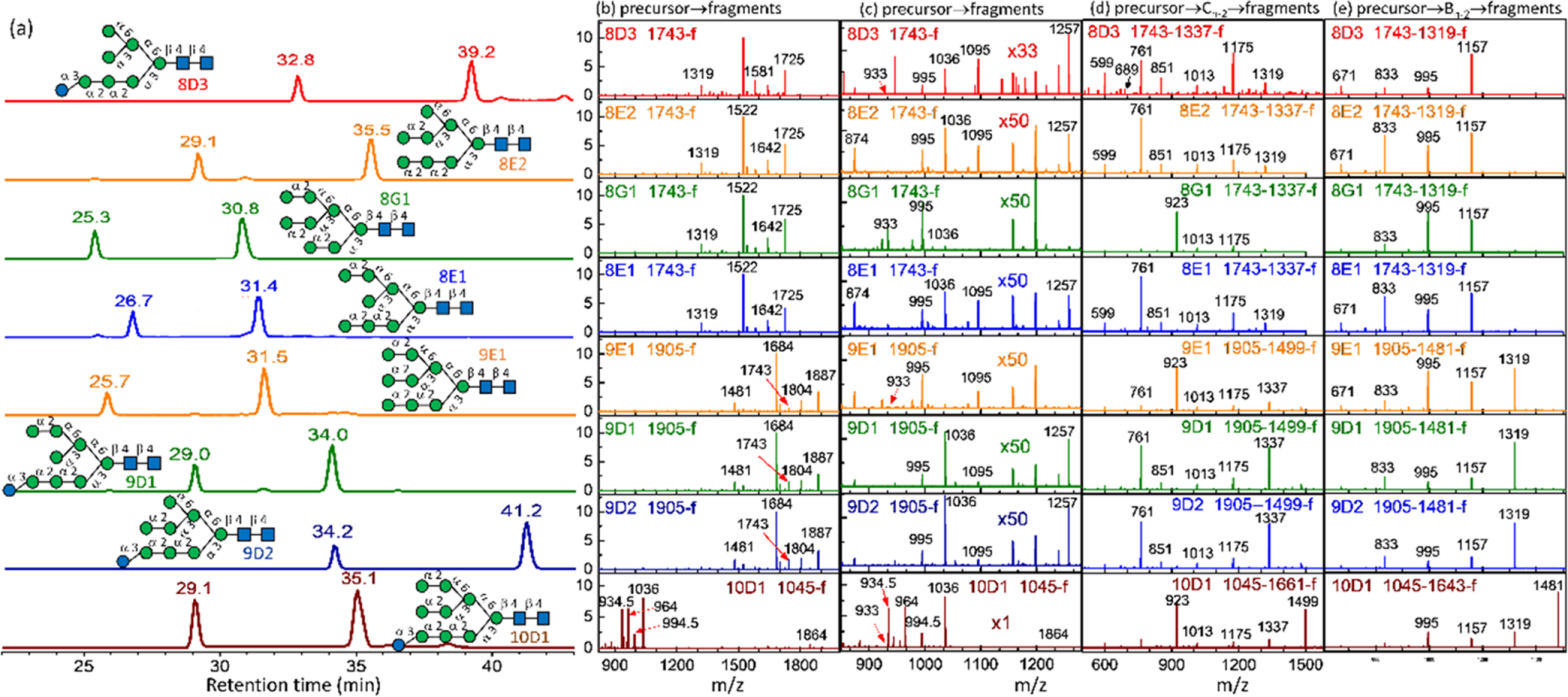
Database of Man_8_GlcNAc_2_, GlcMan_7_GlcNAc_2_, Man_9_GlcNAc_2_, GlcMan_8_GlcNAc_2_, and GlcMan_9_GlcNAc_2_. (a) Chromatogram. CID spectra of sodium ion adducts based on the CID sequence of (b) and (c) precursor → fragments, (d) precursor → C_*n*–2_ ion → fragments and (e) precursor → B_*n*–2_ ion → fragments. The CID sequences are also listed at the top of CID spectra. All *y-*axes represent intensity in arbitrary units. 8E1, 8E2, and 8G1 were extracted from bovine lactoferrin; 8D3 was extracted from hen egg yolk or produced by degradation of 9D1 and 9D2 by enzyme. 9E1 was extracted from bovine lactoferrin; 9D1, 9D2, and 10D1 were extracted from hen egg yolk.

Three isomers of Man_8_GlcNAc_2_, namely, 8E1, 8E2, and 8G1, were extracted from bovine lactoferrin. One isomer of GlcMan_7_GlcNAc_2_, 8D3, was extracted from hen egg yolk. The retention time and MS^2^ spectrum of isomer 8D3 were cross-checked through the degradation of the GlcMan_8_GlcNAc_2_ isomers 9D1 and 9D2 by α-mannosidase from *C. ensiformis*. The chromatograms and CID spectra of these isomers are illustrated in Figure 12. The complete CID spectra are illustrated in (C) CID spectra in the Supporting Information. In the chromatograms, all isomers showed adequate separation through the PGC column and could be easily identified based on the retention time.

CID of the C_*n*–2_ ion occurred through the retro-aldol reaction, as illustrated by the relative intensities of ions with *m*/*z* values of *m*/*z* 761 and 923 in Figure 12d. Easy elimination of Glc from isomer 8D3 was observed; a relatively high intensity was found for fragment ions with *m*/*z* values of 1581, 1175, and 1157 in the 8D3 CID spectra in Figure 12b,d,e respectively.

#### Hex_9_GlcNAc_2_ and Hex_10_GlcNAc_2_

The chromatograms and CID spectra of an Man_9_GlcNAc_2_ isomer (extracted from bovine lactoferrin) and two GlcMan_8_GlcNAc_2_ isomers (extracted from hen egg yolk) are illustrated in Figure 12. The complete CID spectra for structural determination are presented in (C) CID spectra in the Supporting Information. These three isomers were adequately separated on the chromatogram and could be accurately assigned simply based on the retention time. Only one GlcMan_9_GlcNAc_2_ isomer was found. Singly charged precursor ion (one sodium ion adduct) is *m*/*z* 2067, which is beyond the mass range of the ion trap. A doubly charged precursor ion (two sodium ion adducts, *m*/*z* 1045) was used in CID. Similar to the other *N*-glycans, the intensities of ions in the CID spectra through C_*n*–2_ ion [Figure 12d] and B_*n*–2_ ion [Figure 12e] followed the retro-aldol reaction and the easy elimination of Glc occurred in GlcMan_8_GlcNAc_2_ and GlcMan_9_GlcNAc_2_.

## Data Availability

Database in digital format is deposited in MassIVE Repository (https://massive.ucsd.edu/ProteoSAFe/datasets.jsp#%7B%22query%22%3A%7B%7D%2C%22table_sort_history%22%3A%22createdMillis_dsc%22%7D), MSV000093666.

## Supporting Information Available

Logically derived sequence for structural determination of paucimannose *N*-glycan Man_3_GlcNAc_2_ (Figure S1); logically derived sequence for structural determination of paucimannose *N*-glycan Man_4_GlcNAc_2_ (Figure S2); logically derived sequence for structural determination of high-mannose *N*-glycan Man_5_GlcNAc_2_ (Figure S3); logically derived sequence for structural determination of high-mannose *N*-glycan Man_6_GlcNAc_2_ (Figure S4); logically derived sequence for structural determination of high-mannose *N*-glycan Man_7_GlcNAc_2_ (Figure S5); logically derived sequence for structural determination of high-mannose *N*-glycan Man_9_GlcNAc_2_ (Figure S6); logically derived sequence for structural determination of high-mannose *N*-glycan Man_10_GlcNAc_2_ (Figure S7); CID spectra of sodium ion adduct for structural determination of *N*-glycan GlcMan_5_GlcNAc_2_ isomer 6H1(Figure S8); CID spectra of sodium ion adduct for structural determination of *N*-glycan GlcMan_6_GlcNAc_2_ isomer 7F1 (Figure S9); CID spectra of sodium ion adduct for structural determination of *N*-glycan Hex_8_GlcNAc_2_ isomer 8E1 (Figure S10); CID spectra of sodium ion adduct for structural determination of *N*-glycan Hex_8_GlcNAc_2_ isomer 8E2 (Figure S11); CID spectra of sodium ion adduct for structural determination of *N*-glycan Hex_8_GlcNAc_2_ isomer 8G1 (Figure S12); CID spectra of sodium ion adduct for structural determination of *N*-glycan Hex_8_GlcNAc_2_ isomer 8D3 (Figure S13); CID spectra of sodium ion adduct for structural determination of *N*-glycan Hex_9_GlcNAc_2_ isomer 9D1 (Figure S14); CID spectra of sodium ion adduct for structural determination of *N*-glycan Hex_9_GlcNAc_2_ isomer 9D2 (Figure S15); CID spectra of sodium ion adduct for structural determination of *N*-glycan Hex_9_GlcNAc_2_ isomer 9E1 (Figure S16); CID spectra of sodium ion adduct for structural determination of *N*-glycan Hex_10_GlcNAc_2_ isomer 10D1 (Figure S17); chromatograms and structure assignments of high-mannose *N*-glycans extracted from human mammary epithelial cells and breast carcinoma (Figure S18); and diagnostic fragments of the isomers with close retention times (Table S1) (PDF)
