# Supplementary material for: Chromatograms and Mass Spectra of High-Mannose and Paucimannose N-Glycans for Rapid Isomeric Identifications
Source: J Proteome Res. 2024 Feb 16;23(3):939–55. doi: 10.1021/acs.jproteome.3c00640 (PMC10913092; doi:10.1021/acs.jproteome.3c00640)
Supplement: Supplementary file 1 — pr3c00640_si_001.pdf [file pr3c00640_si_001.pdf]

Supporting Information

Chromatograms and mass spectra of high-mannose and paucimannose *N*-glycans for rapid isomeric identifications

Chia Yen Liew[1,2,3], Jien-Lian Chen[1], Yen-Ting Lin[1], Hong-Sheng Luo[1,4], An-Ti Hung[1,5], Bryan John Abel Magoling[1,6,7], Hock-Seng Nguan[1], Charles Pin-Kuang Lai[1,7,8], Chi-Kung Ni*[1,3,5]

[1] Institute of Atomic and Molecular Sciences, Academia Sinica, Taipei 106216, Taiwan

[2] International Graduate Program of Molecular Science and Technology, National Taiwan University, Taipei 106216, Taiwan

[3] Molecular Science and Technology, Taiwan International Graduate Program, Academia Sinica, Taipei 106216, Taiwan.

[4] Department of Chemistry, National Taiwan Normal University, Taipei 116059, Taiwan

[5] Department of Chemistry, National Tsing Hua University, Hsinchu 300044, Taiwan

[6] Institute of Biochemical Sciences, College of Life Science, National Taiwan University, Taipei 106216, Taiwan

[7] Chemical Biology and Molecular Biophysics Program, Taiwan International Graduate Program, Academia Sinica, Taipei 115201, Taiwan

[8] Genome and Systems Biology Degree Program, National Taiwan University and Academia Sinica, Taipei 106216, Taiwan

*Corresponding authors, e-mail addresses: ckni@po.iams.sinica.edu.tw

Contents

A. Sources of materials

B. Logically derived sequences

C. CID spectra

D. Diagnostic fragments

E. Conversion from retention time to dextran index

F. Chromatograms of human breast carcinoma

A. Source of materials

The *N*-glycans, ManGlcNAc$_2$, 2E1, 2F1, 3D1, 3F1, 4D1, 4D2, 4D3, 4E1, 4E2, 4E3, 4F1, 5D1, 5D2, 6E1, 6E2, 6G1, 7D1, and 7D2 were purchased from Omicron Biochemicals, Inc. (South Bend, IN, USA). Dextran *Leuconostoc Mesenteroides* (analytical standard, for GPC, Mw 1,000) was purchased from Sigma Aldrich; bovine lactoferrin and bovine whey protein were purchased from AOR Inc. (Clifton, NJ, USA). Soybeans, black beans, hen egg yolk powder, and hen eggs were purchased from local market.

Enzyme α-mannosidase of *Canavalia ensiformis* (Jack bean) and α1-6 mannosidase, and PNGase F was purchased from New England Biolabs (Ipswich, MA, USA). All the cell lines were gifted by Dr. Ruey-Hwa Chen, Institute of Biological Chemistry, Academia Sinica. IgY purification kits was purchased from Gallus Immunotech Inc. (Nanterre, France).

The C18 cartridges were purchased from Waters (Milford, MA, USA), and NPGC cartridges were purchased from Extract-Clean SPE Carbo, Grace (Columbia, MD, USA). TSKgel Amide-80 columns (150 mm × 2.0 mm, particle size of 5 µm) were purchased from Tosoh Bioscience GmbH (Griesheim, Germany), PGC Hypercarb columns (2.1 mm × 150 mm or 2.1 mm × 100 mm, particle size of 3 µm) were purchased from Thermo Fisher Scientific (Waltham, MA, USA).

B. Logically derived sequences

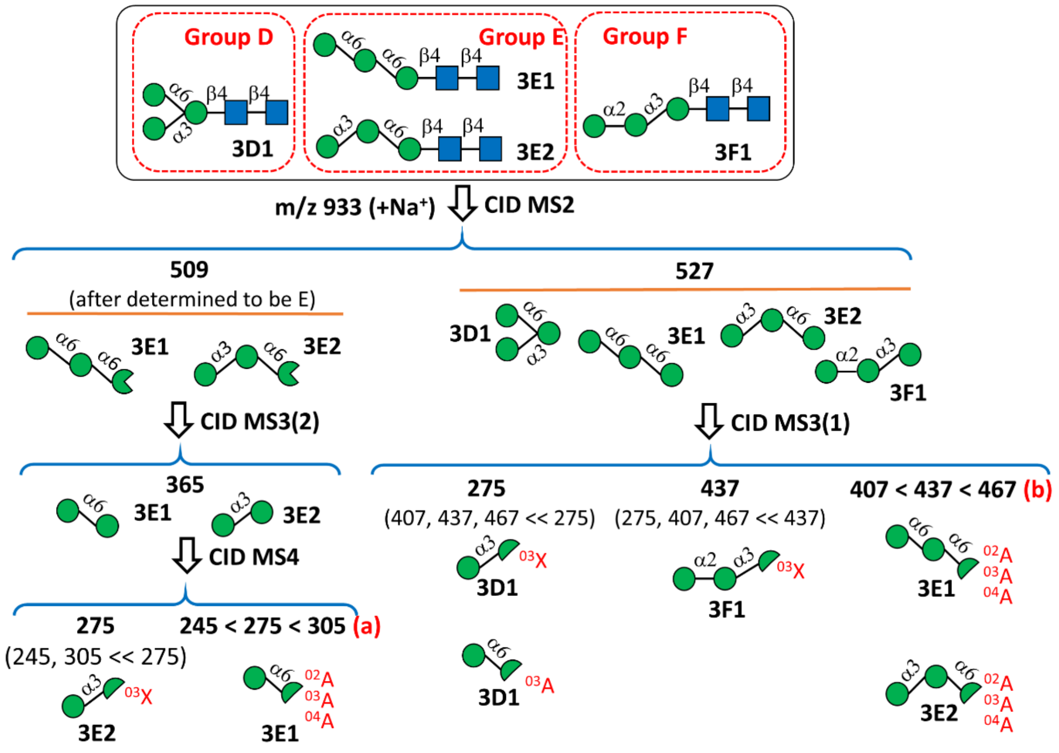

(a) Intensity ratios of ions m/z 305, 275, and 245 are 5 : 3±1 : 1±0.5.
(b) Intensity ratios of ions m/z 467, 437, and 407 are 5 : 3±1 : 1±0.5.

Fig. S1 Logically derived sequence for structural determination of paucimannose *N*-glycan $Man_3GlcNAc_2$ sodium ion adducts.

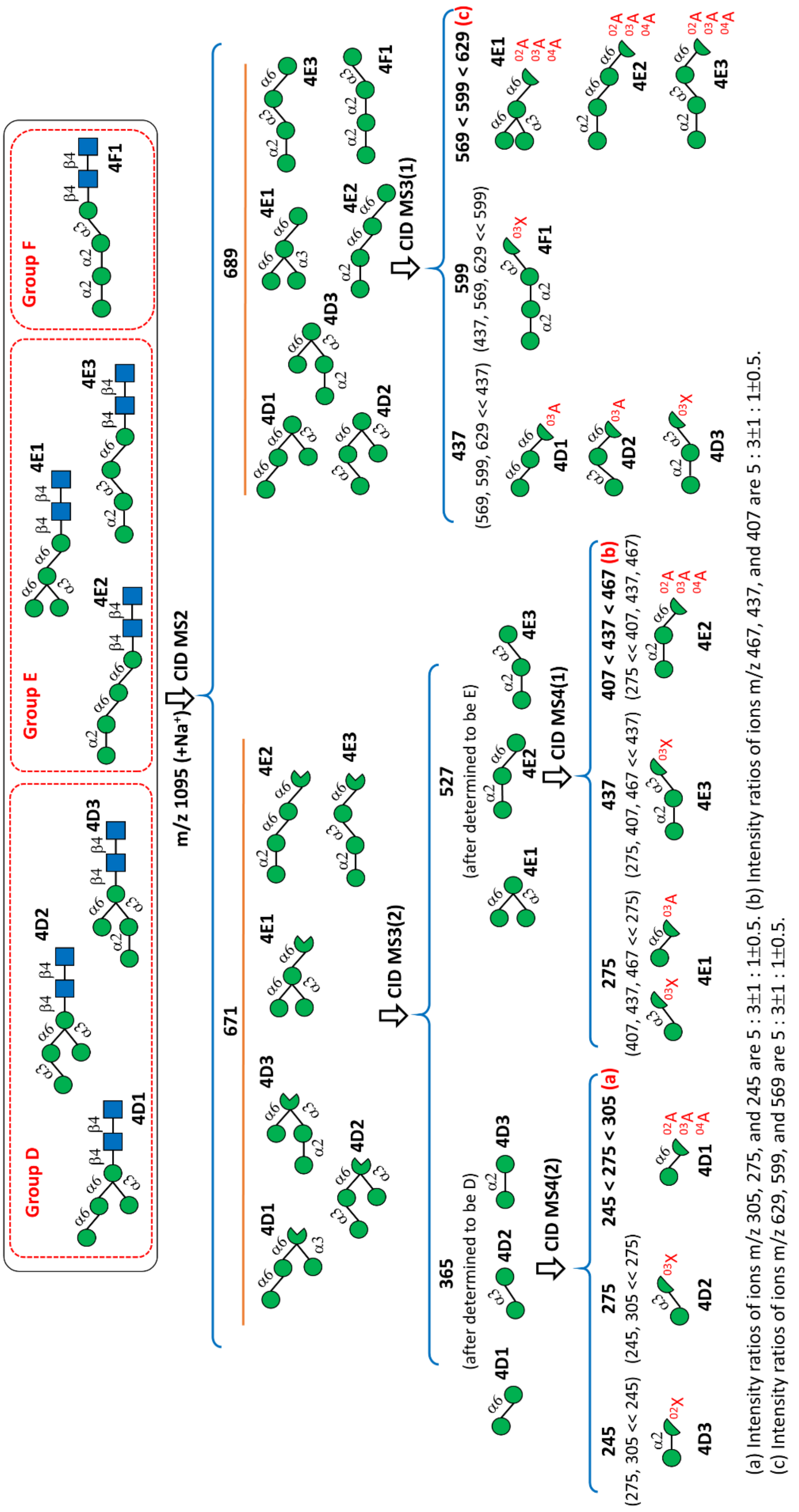

Fig. S2 Logically derived sequence for structural determination of paucimannose *N*-glycan $Man_4GlcNAc_2$ sodium ion adducts.

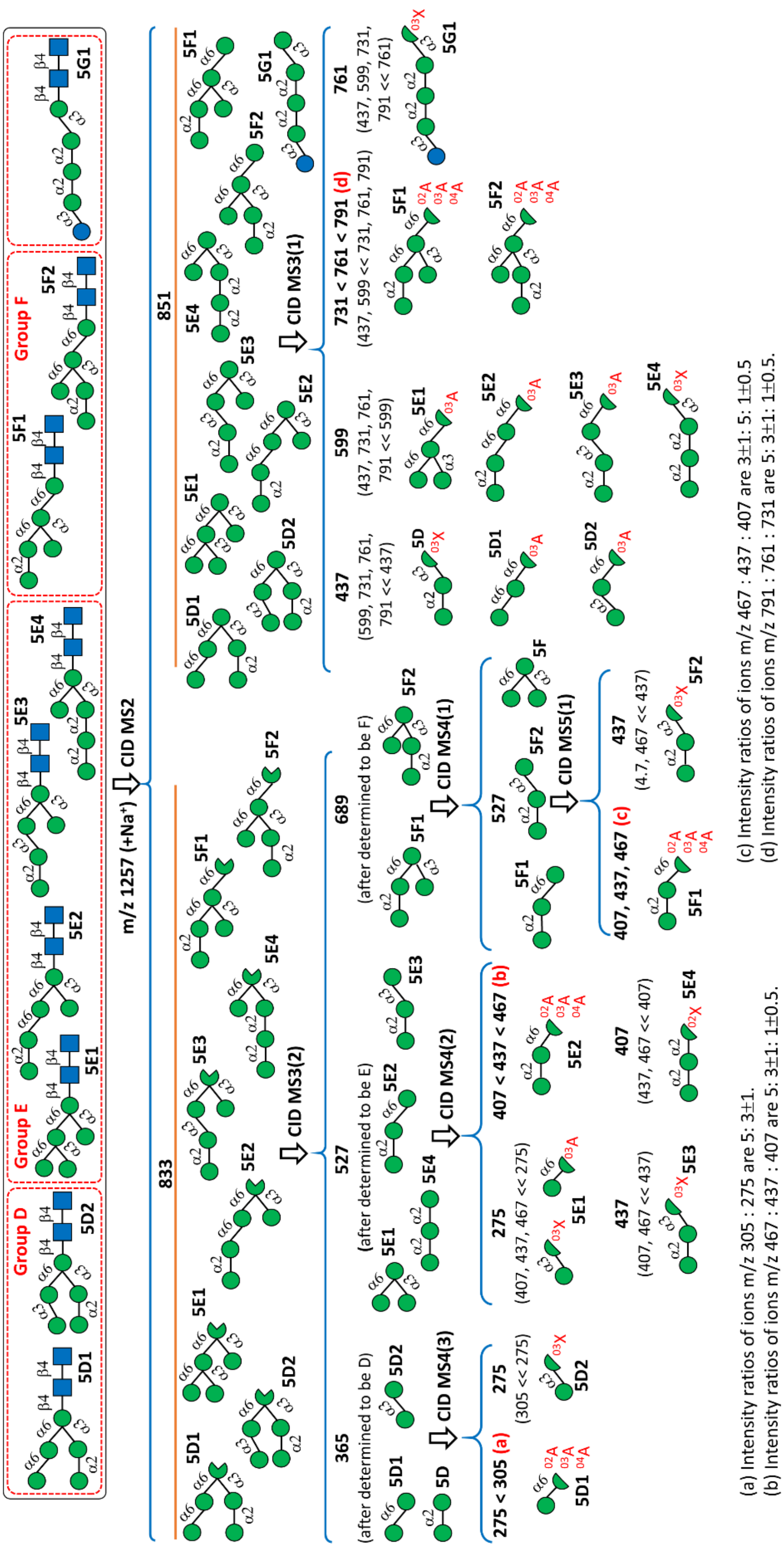

Fig. S3 Logically derived sequence for structural determination of highmannose *N*-glycan $Hex_5GlcNAc_2$ sodium ion adducts.

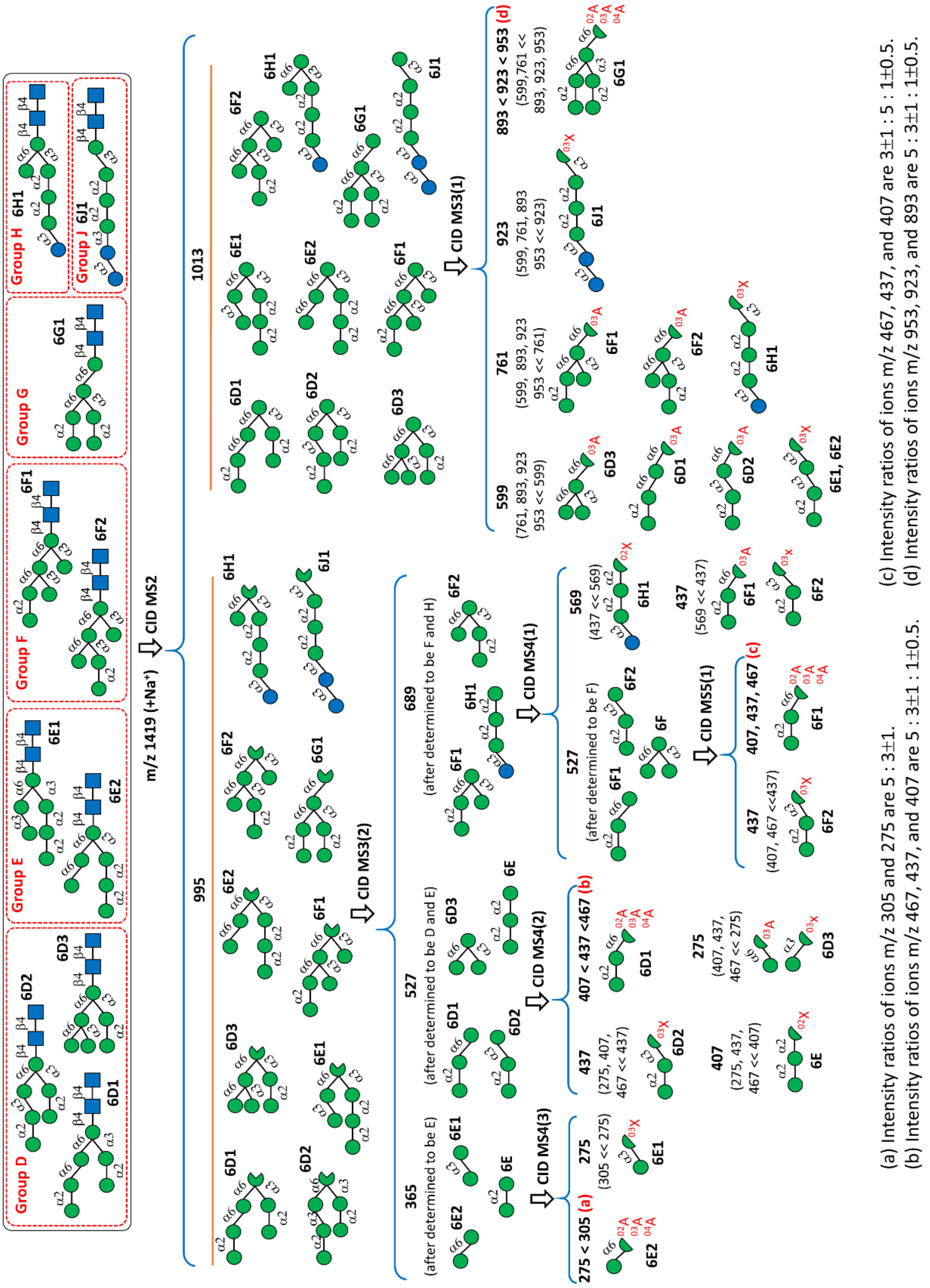

Fig. S4 Logically derived sequence for structural determination of highmannose *N*-glycan $Hex_6GlcNAc_2$ sodium ion adducts.

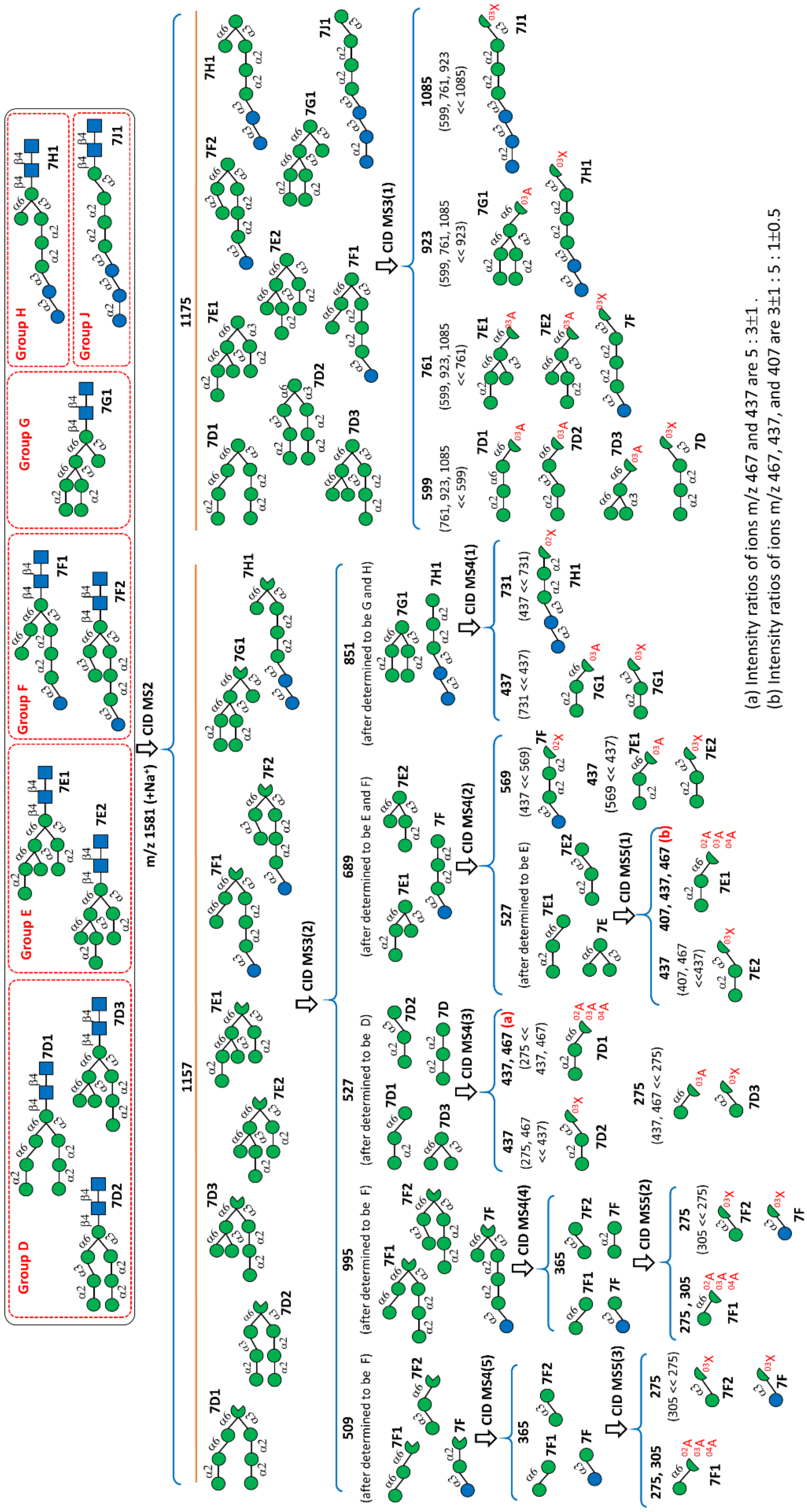

Fig. S5 Logically derived sequence for structural determination of highmannose *N*-glycan $Hex_7GlcNAc_2$ sodium ion adducts.

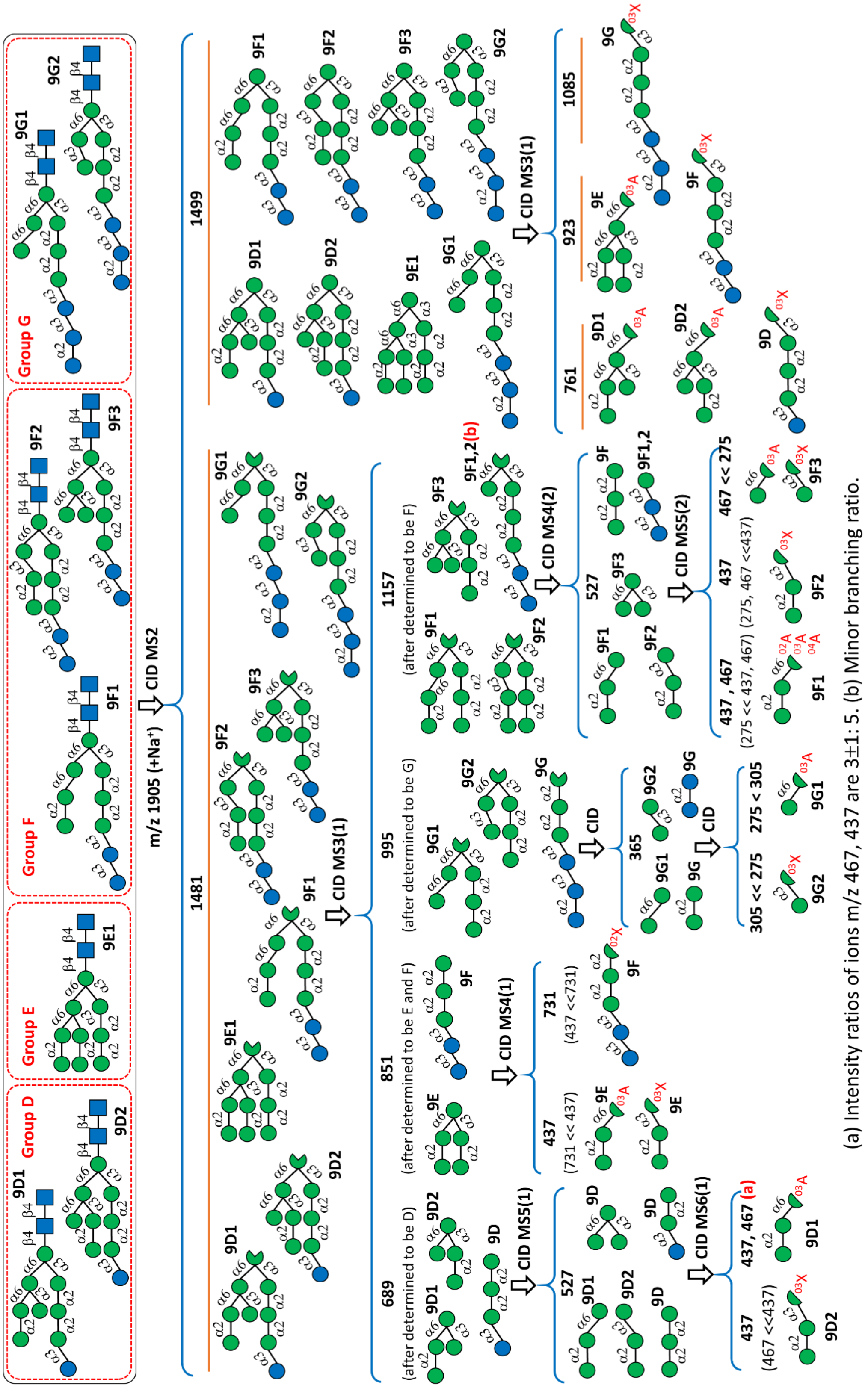

Fig. S6 Logically derived sequence for structural determination of highmannose *N*-glycan $Hex_9GlcNAc_2$ sodium ion adducts.

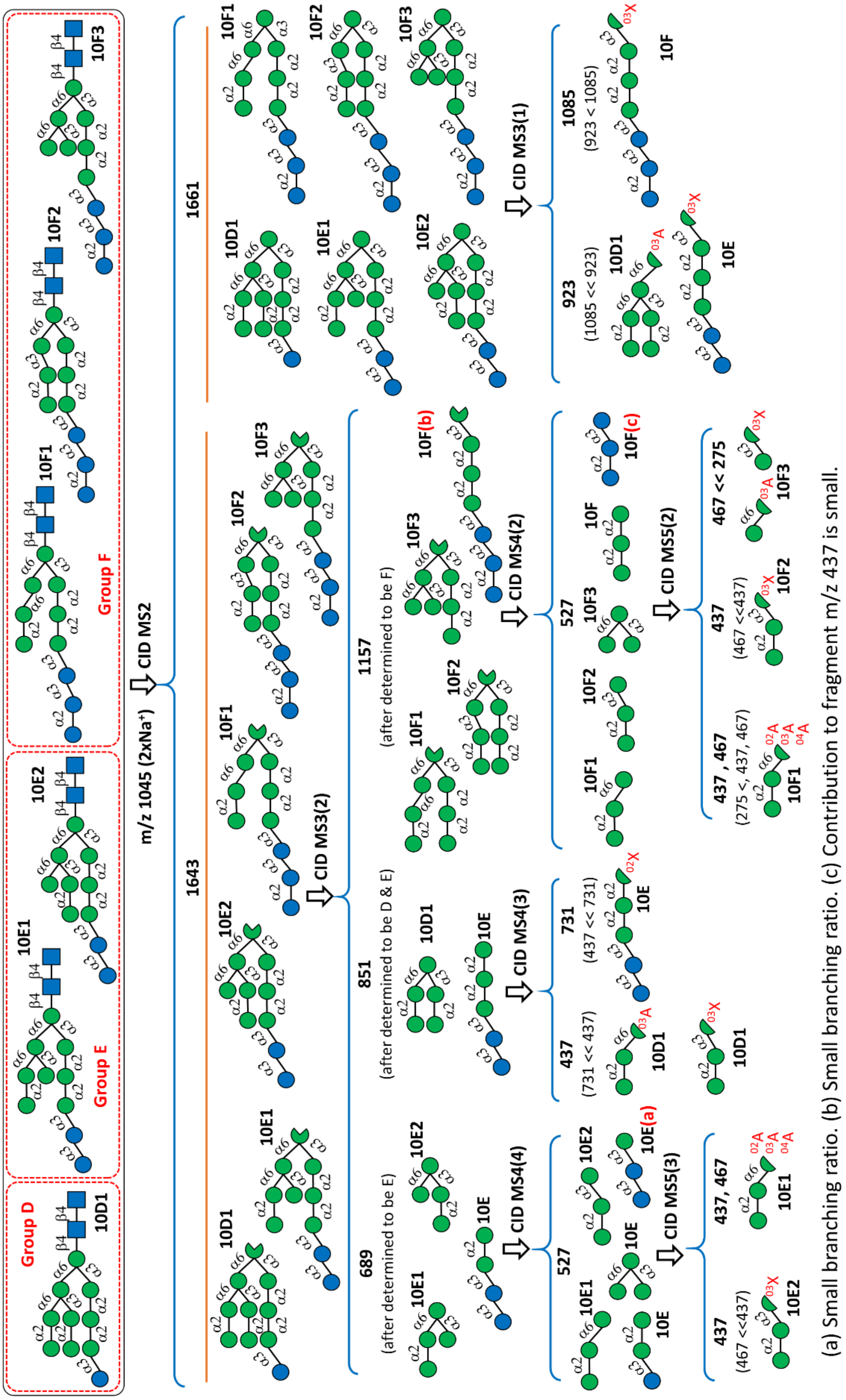

Fig. S7 Logically derived sequence for structural determination of highmannose *N*-glycan $Hex_{10}GlcNAc_2$ sodium ion adducts.

C. CID spectra

The CID spectra of database of $Man_nGlcNAc_2$, n=1 to 4, are illustrated in main text. The CID spectra of database of $Man_5GlcNAc_2$ were illustrated in our previous report.[36] Parts of the CID spectra of database of $Hex_nGlcNAc_2$, n=6 and 7, were illustrated in our previous report,[36] rest of the CID are illustrated in Fig. S8 and Fig. S9. The complete CID spectra for structural determination of $Hex_5GlcNAc_2$, n=8-10, are illustrated in Fig. S10 and Fig. S17.

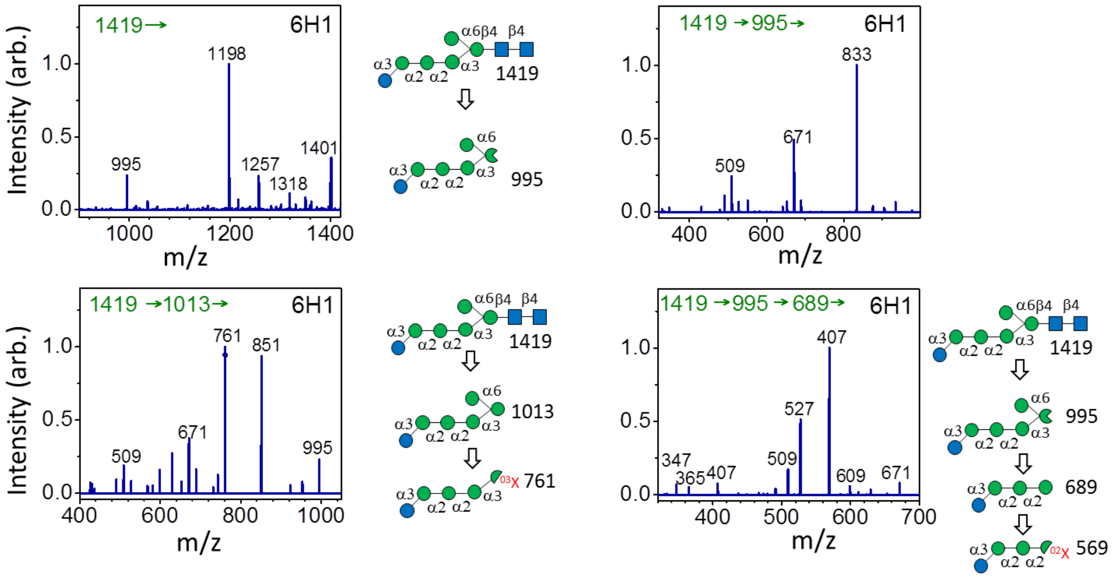

Fig. S8 CID spectra of sodium ion adduct for structural determination of *N*-glycan $GlcMan_5GlcNAc_2$ isomer 6H1.

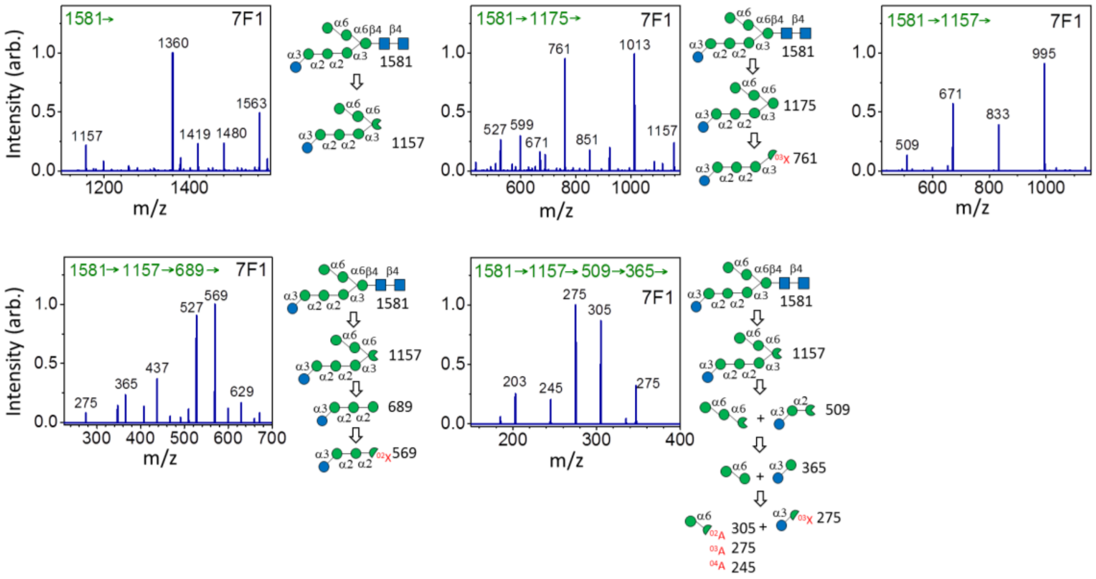

Fig. S9 CID spectra of sodium ion adduct for structural determination of *N*-glycan $GlcMan_6GlcNAc_2$ isomer 7F1.

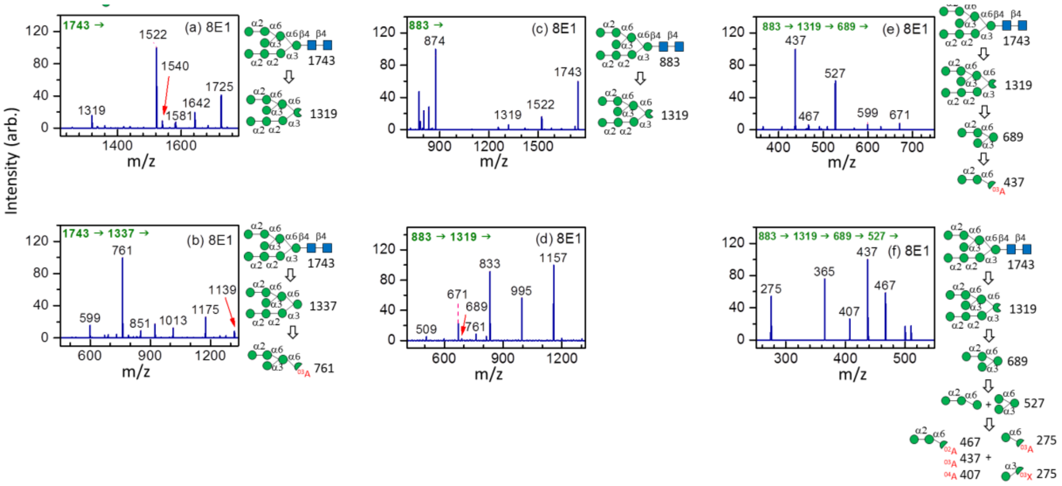

Fig. S10. CID spectra of sodium ion adducts for structural determination of *N*-glycans $Hex_8GlcNAc_2$, isomer 8E1.

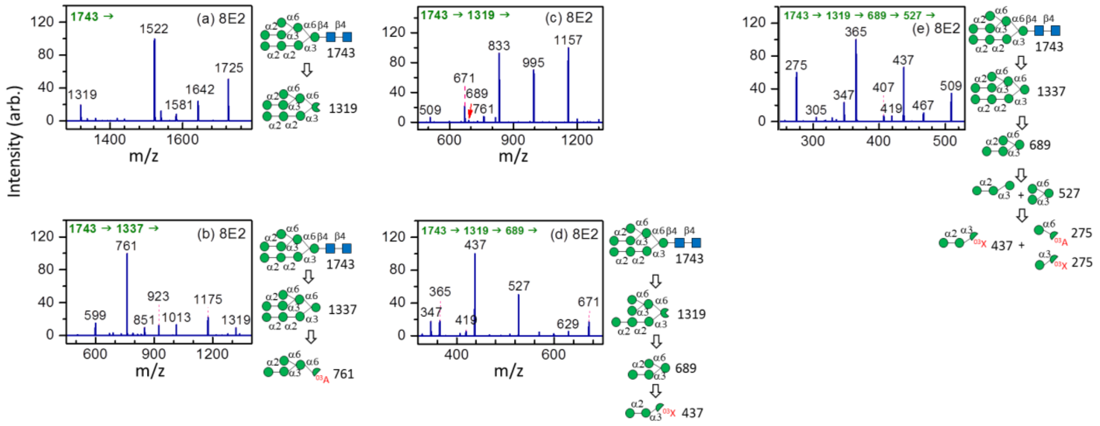

Fig. S11. CID spectra of sodium ion adducts for structural determination of *N*-glycans $Hex_8GlcNAc_2$, isomer 8E2.

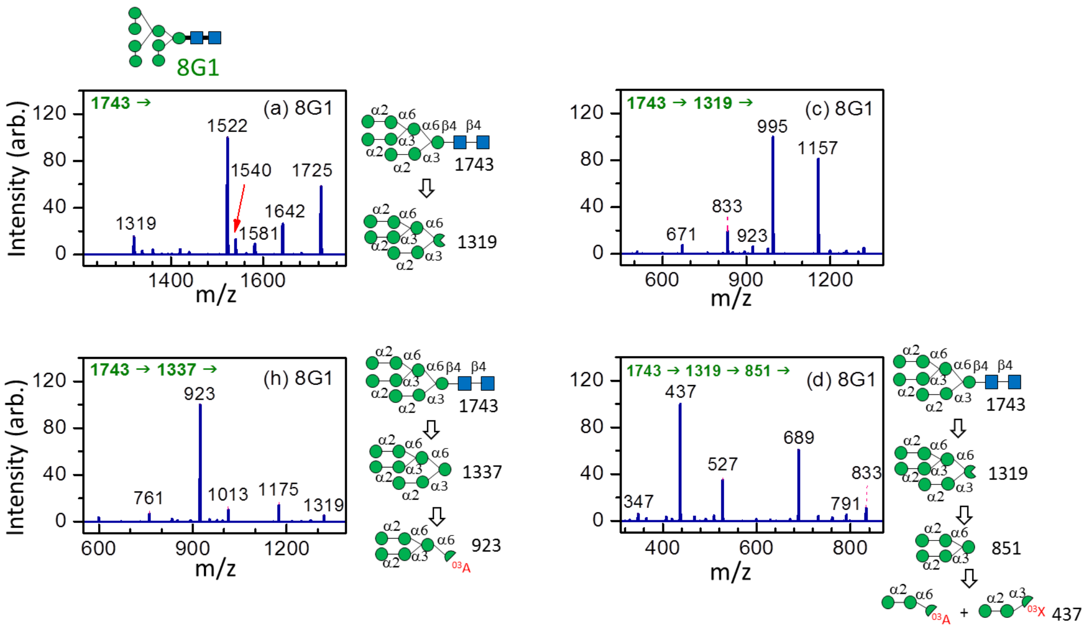

Fig. S12. CID spectra of sodium ion adducts for structural determination of *N*-glycans $Hex_8GlcNAc_2$, isomer 8G1.

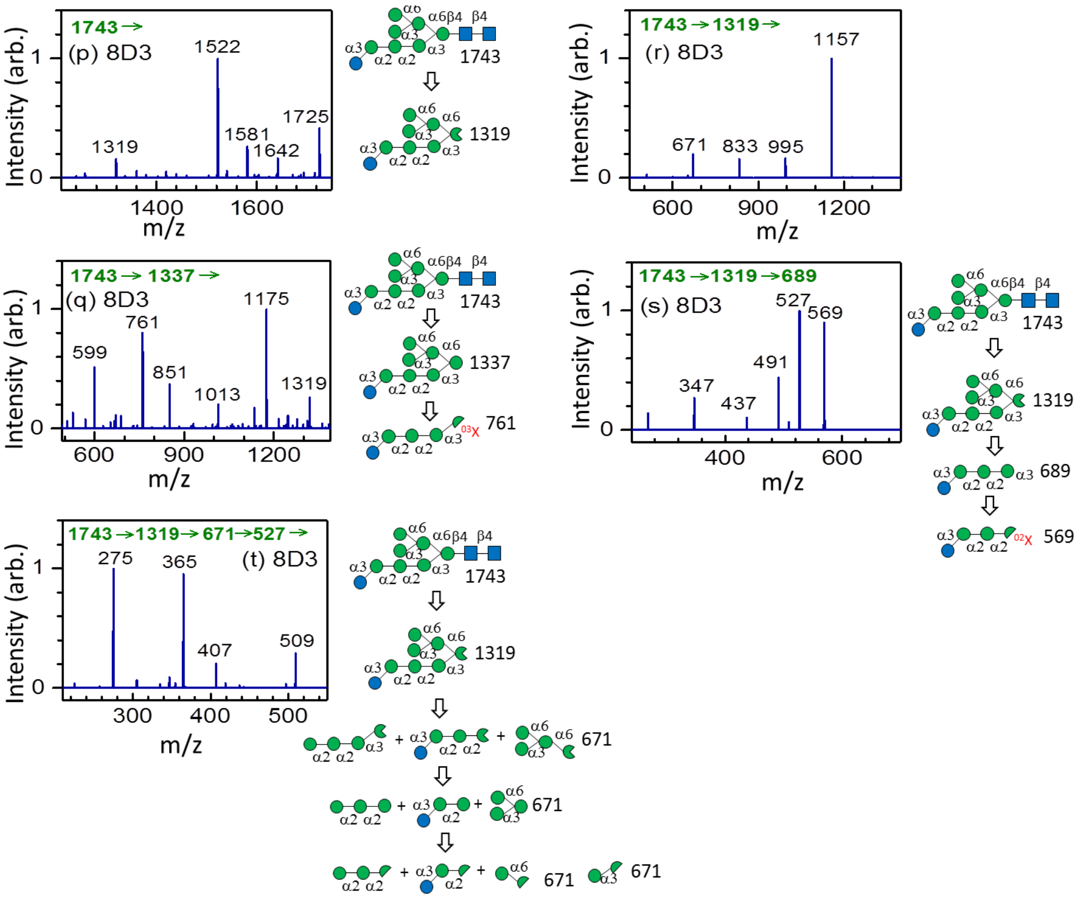

Fig. S13. CID spectra of sodium ion adducts for structural determination of *N*-glycans $Hex_8GlcNAc_2$, isomer 8D3.

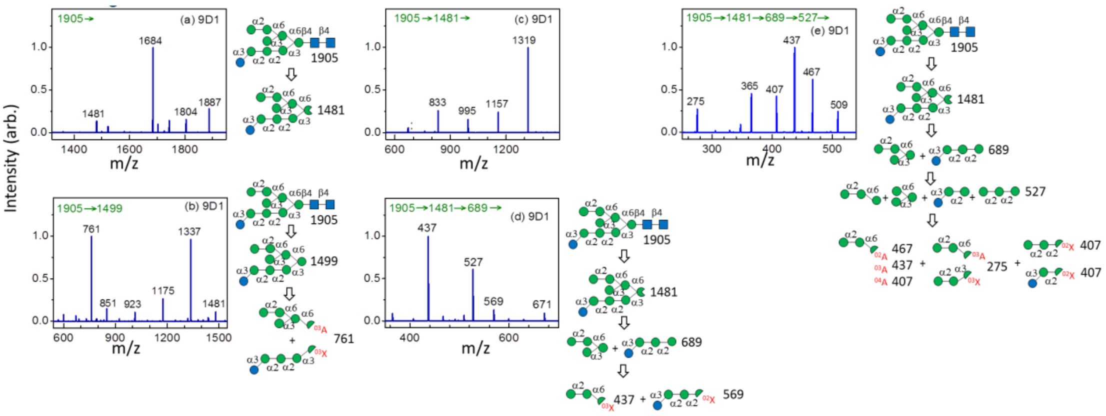

Fig. S14. CID spectra of sodium ion adducts for structural determination of *N*-glycans $Hex_9GlcNAc_2$, isomer 9D1.

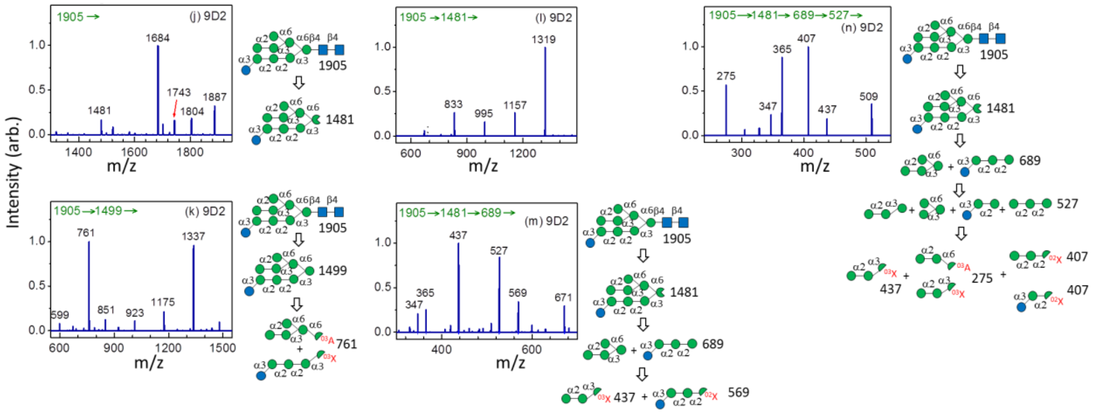

Fig. S15. CID spectra of sodium ion adducts for structural determination of N-glycans $Hex_9GlcNAc_2$, isomer 9D2.

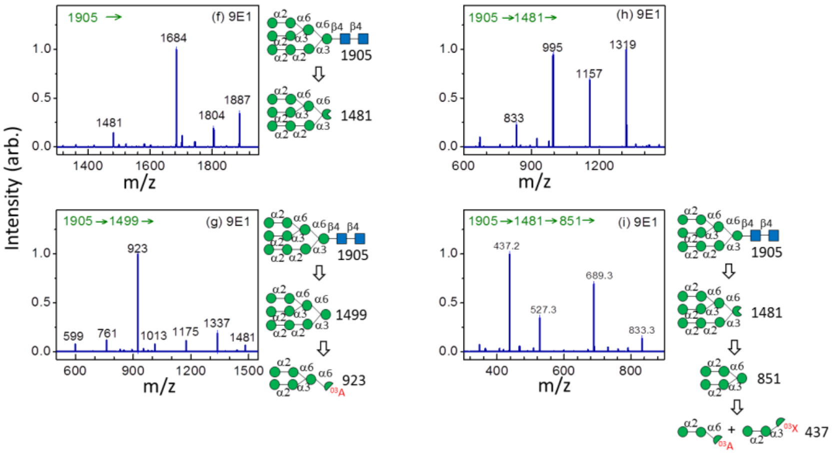

Fig. S16. CID spectra of sodium ion adducts for structural determination of N-glycans $Hex_9GlcNAc_2$, isomer 9E1.

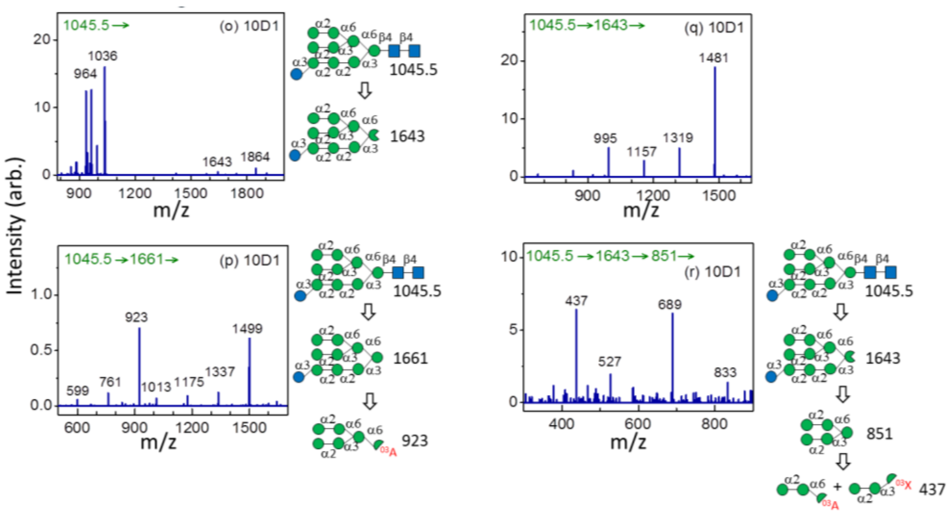

Fig. S17. CID spectra of sodium ion adducts for structural determination of N-glycan $Hex_{10}GlcNAc_2$, isomer 10D1.

D. Diagnostic fragments

Table S1. Diagnostic fragments of the isomers with close retention times.

N-glycan	MS2 (large peaks)	MS2 (small peaks)	MS3 C ion	MS3 B ion	MS4
**Man4**	**1257-f**	**1095-f**	**1095-689-f**	**1095-671-f**	**1095-671-365-f or 1095-365-f**
4E1		550, 609, 771			275, 347, 365, 407, 437, 467
4E2		550, 609, 771			275, 347, 365, 407, 437, 467
**Man5**	**1257-f**	**1257-f**	**1257-851-f**	**1257-833-f**	**1257-833-527 or 1257-527-f**
5E4		609, 671, 771	599, 731, 761, 791	509, 671, 689, 761	
5E2		671, 712, 933			275, 365, 467
5E1		671, 712, 933			275, 365, 467
5F1		609, 671, 771,	599, 731, 761, 791	509, 671, 689, 761	
**Man6**	**1419-f**	**1419-f**	**1419-1013-f**	**1419-995-f**	**1419-995-527-f or 1419-527-f**
6H1	1257, 1318	712, 771, 933	599, 761, 851,	509, 671, 833,	
6F1	1257, 1318	712, 771, 933	599, 761, 851,	509, 671, 833,	
6D3	1257, 1318	712, 771, 933	599, 761, 851,	509, 671, 833,	275, 365, 407
6E2	1257, 1318	712, 771, 933	599, 761, 851,	509, 671, 833,	275, 365, 407
**Man7**	**1581-f**	**1581-f**	**15819-f**	**1581-1175-f**	**1581-1157-527-f or 1581-527-f**
7F1	1419, 1480,	1419, 1480	771, 833, 874, 933, 1095	599, 761 , 1013	
7D3	1419, 1480,	1419, 1480	771, 833, 874, 933, 1095	599, 761 , 1013	275, 437, 467
7D1	1419, 1480,	1419, 1480	771, 833, 874, 933, 1095	599, 761 , 1013	275, 437, 467

E. Conversion from retention time to dextran index

If the retention time of the analyte is located between the dextran peak n and n+1, then the conversion of the analyte retention time to the index based on the retention time of dextran is calculated using the following equation.

$$\text{Index(analyte)} = n + \frac{RT(analyte) - RT(Dextran\ of\ nth\ peak)}{RT(Dextran\ of\ (n+1)th\ peak) - RT(Dextran\ of\ nth\ peak)}$$

Where RT(analyte), RT(Dextran of nth peak), and RT(Dextran of (n+1)th peak) represent the HPLC retention time of analyte, nth peak of dextran, n+1 peak of dextran. The sequences of the dextran peaks in chromatogram are illustrated in main text. The numberings of dextran peaks start from ions m/z 365 (n=1 and 2), m/z 527 (n=3 and 4), m/z 689 (n=5 and 6), m/z 851 (n=7 and 8), m/z 1013 (n=9, 10), m/z

1175 (n=11 and 12), m/z 1337 (n=13 and 14). Notably, dextran is not reduced. For each isomer, there are two major peaks, corresponding to α and β anomers of the sugar at the reducing end of linear isomers, whereas the minor peaks, corresponding to branched isomers, are not taken into consideration.

F. Chromatograms of human breast carcinoma

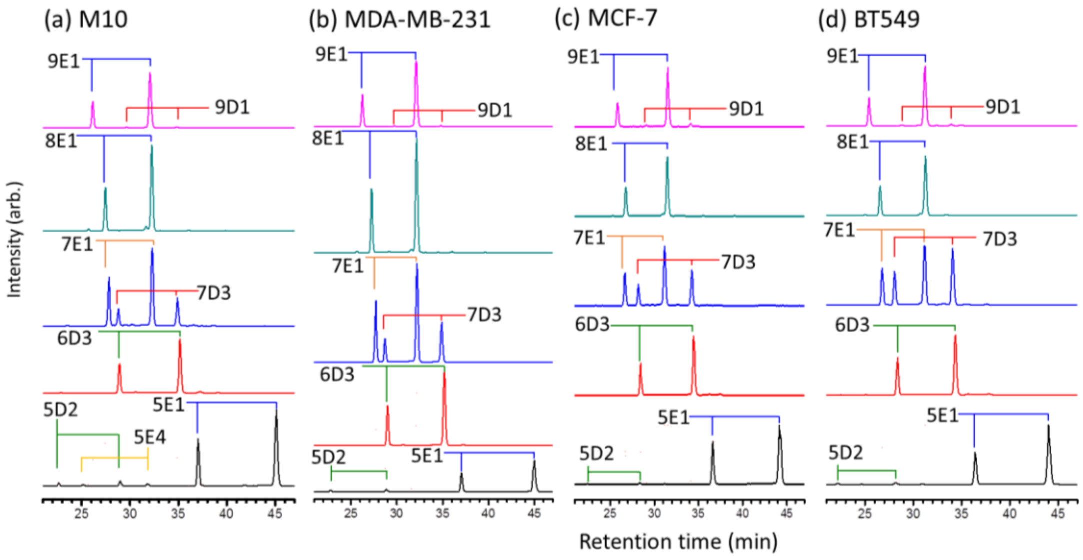

Fig. S18 Chromatograms and structure assignments of high mannose *N*-glycans extracted from human mammary epithelial cells and breast carcinoma.
